# Dual-Site Inhibition of SARS-CoV‑2 RNA-Dependent RNA Polymerase by Small Molecules Able to Block Viral Replication Identified through a Computer-Aided Drug Discovery Approach

**DOI:** 10.1021/acsinfecdis.5c00517

**Published:** 2025-09-26

**Authors:** Paolo Malune, Daniela Iaconis, Candida Manelfi, Stefano Giunta, Roberta Emmolo, Filippo Lunghini, Annalaura Paulis, Carmine Talarico, Angela Corona, Andrea Rosario Beccari, Enzo Tramontano, Francesca Esposito

**Affiliations:** † Department of Life and Environmental Sciences, 204509 University of Cagliari, Cittadella Universitaria di Monserrato, Cagliari 09124, Italy; ‡ EXSCALATE, 18798 Dompé farmaceutici S.p.A., Via Tommaso De Amicis, 95, Napoli 80131, Italy

**Keywords:** SARS-CoV-2, RNA-dependent RNA polymerase, nsp12, drug discovery, drug repurposing, CADD

## Abstract

Since its emergence in late 2019, SARS-CoV-2, the causative agent of COVID-19, has continued to spread globally, with more than 7 million reported deaths as of March 2025. Among the viral nonstructural proteins, nsp12 serves as the RNA-dependent RNA polymerase (RdRp), mediating viral genome replication and transcription in concert with its cofactors nsp7 and nsp8. To date, only two nucleoside analogs specifically targeting SARS-CoV-2 nsp12, remdesivir and molnupiravir, have been authorized by the FDA for COVID-19 treatment. In response to the need for additional safe and effective antiviral agents, we screened two extensive in silico libraries of safe-in-man compounds (>9,000) and natural compounds (>249,000), against the SARS-CoV-2 nsp12/7/8 complex, targeting the orthosteric and two allosteric nsp12 sites, using the EXSCALATE (EXaSCale smArt pLatform Against paThogEns) platform. Compounds were then selected based on docking score significance, novelty for the target, and clinical safety profiles. The top 119 candidates were subsequently evaluated in a biochemical assay to assess their potential to inhibit SARS-CoV-2 nsp12/7/8 polymerase activity, identifying 42 compounds able to block it, among which four showed IC_50_ and EC_50_ values in the nanomolar or low micromolar range. When tested in cell-based assays to evaluate their efficacy on SARS-CoV-2 replication, they proved to inhibit it in the same concentration ranges. Mechanism of action studies revealed different modalities of inhibition. These results provide the basis for the development of novel antiviral compounds against SARS-CoV-2, targeting both the RdRp active site and an allosteric site, further suggesting that the Computer-Aided Drug Discovery (CADD) approach, together with experimental validation, can provide the basis for accelerated antiviral drug development.

## Graphical abstract

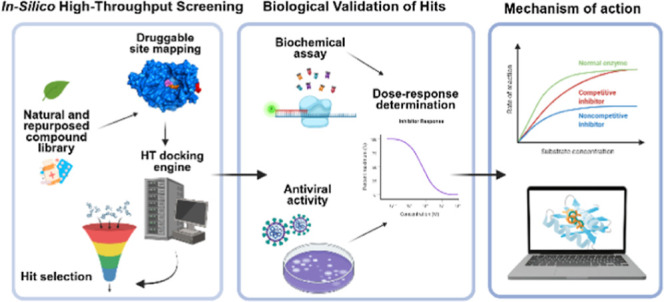

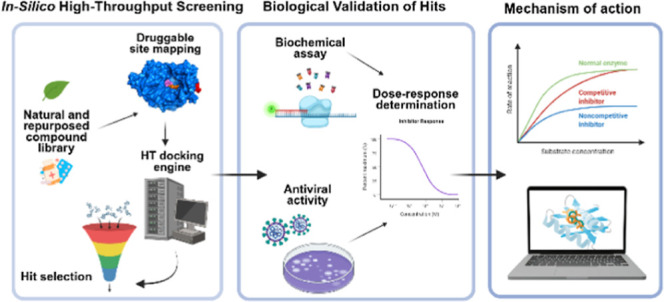

Five years after its emergence in late 2019, SARS-CoV-2 remains a global threat and a major human pathogen.^1,2^ Despite remarkable and successful scientific efforts to develop effective antivirals and vaccines, the virus continues to spread with more than 778 million confirmed COVID-19 cases and over 7 million COVID-19-related deaths, as of September 2025.^3^ In addition to the clinical, social, and economic burdens given by the widespread acute infection, approximately 1 in 3 people reports “long COVID”, with persisting symptoms even three months after SARS-CoV-2 infection.^4^ Among the most attractive targets in drug development to block viral replication, viral polymerases have a central role, and, in fact, a significant portion of the antiviral drugs approved so far targets this enzymatic function, with either nucleoside/pyrophosphate analogs or non-nucleotide inhibitors.^5^ Moreover, RNA-dependent RNA polymerases (RdRp) are the most conserved proteins in the viral RNA world, with a preserved right-hand tertiary structure and common target-binding features and amino acid residues.^6^ SARS-CoV-2, possessing one of the largest known RNA genome, encodes for 16 nonstructural, 4 structural, and several accessory proteins.^7^ Among the SARS-CoV-2 nonstructural proteins, nsp12 is the viral RdRp, involved in the crucial tasks of genome replication, discontinuous transcription of subgenomic mRNAs, and “backtracking” on the template/primer complex to allow proofreading activity in the presence of mismatches.^8^ The nsp12 possesses Nidovirus RdRp-associated nucleotidyl transferase (NiRAN) and RdRp domains, the latter of which designs the classical right-hand shape that is typical of RNA and DNA polymerases, with three conserved subdomains, Palm, Thumb, and Fingers. Nsp12 acts in complex with cofactors nsp7 and nsp8, with which it forms the minimal replication–transcription complex (RTC).^8^ To date (September 2025), only three drugs, remdesivir (Veklury), molnupiravir (Lagevrio), and nirmatrelvir (Paxlovid), reached clinical use for COVID-19 treatment under FDA approval or authorization under emergency use.^9^ Among these, two (molnupiravir and remdesivir) target SARS-CoV-2 nsp12 with different mechanisms of action. Remdesivir (or GS-5734), developed in 2017 to fight Ebola virus infection, inhibits nsp12 by direct interference on RdRp enzymatic activity as a delayed chain-terminator,^10^ preventing UTP incorporation in the nascent strand when incorporated as an ATP analog into the template RNA.^11,12^ Molnupiravir, in contrast, does not block nsp12 processivity and it is instead incorporated into the nascent RNA strand as G or A, inducing lethal mutagenesis by a strong increase in the frequency of purine transitions.^13^ However, the efficacy of treatment with remdesivir is still under evaluation and requires further data,^14–16^ while molnupiravir has been discontinued in Europe after the developing pharmaceutical company, Merck Sharp and Dohme, withdrew the application for marketing authorization to the European Medicines Agency (EMA), subsequently to the evaluation of the clinical data by the Committee for Medicinal Products for Human Use not concluding positively on the risk–benefit balance for this drug.^17^ In addition, molnupiravir has been shown to favor the emergence of novel SARS-CoV-2 variants by increasing mutation rates, as demonstrated by the finding of molnupiravir-linked mutational signatures in circulating viruses from areas and age groups associated with widespread use of the drug.^18^ These findings strengthen the use of the viral RdRp as a drug target and indicate the need for the identification of new inhibitors to effectively fight SARS-CoV-2, as well as other human Coronaviruses.

In the present work, with the aim of identifying novel SARS-CoV-2 nsp12 inhibitors, we exploited advanced computational docking protocols developed to screen and select promising non-nucleos­(t)­ide candidates. The in silico predictions, exploiting two libraries of repurposed compounds and natural deriving compounds, were then validated through a series of biochemical and viral replication assays, allowing confirmation of both the robustness of the predictions and the antiviral efficacy of these novel anti-SARS-CoV-2 compounds. In addition to leveraging structurally and pharmacologically diverse scaffolds, the use of commercialized or under active development compounds offers the advantage of focusing on a repurposing strategy, exploiting molecules with known bioactivity and rigorous testing and safety assessments, thus accelerating the drug discovery process of novel antiviral compounds against SARS-CoV-2. Additionally, we screened a library of naturally derived compounds, reflecting the increasing interest in natural products and their recognized potential to provide a source of bioactive molecules with inhibitory activity against viral nonstructural proteins,^19,20^ combined with significant research efforts devoted to studying and classifying biodiversity. In the present work, we identified four compounds active against SARS-CoV-2 replication, in addition to several chemically diverse molecules active against SARS-CoV-2 RdRp function, which can potentially provide the structural basis for the development of more potent and safer antivirals.

## Results and Discussion

### In Silico Docking of Compounds against SARS-CoV-2 RTC

With the aim of identifying novel SARS-CoV-2 RdRp inhibitors, two libraries were screened with a molecular docking protocol against the RTC assembly of SARS-CoV-2, a first library of 10,000 pharmaceutical compounds (Safe-In-Man collection), for a repurposing approach, and a second library of ∼250.000 natural compounds. The cryo-EM structure of the SARS-CoV-2 nsp12, nsp8, and nsp7 complex was retrieved from the Protein Data Bank (PDB: 7BV2), and three nsp12 sites were used as target sites of the docking prediction: the active (orthosteric) site and two allosteric sites (from now on identified as Palm and Thumb). The protein crystal structure was complexed with remdesivir, which was used as the binding mode reference. The in silico docking allowed having a total of 27,000 poses obtained from 9,033 repurposed compounds and around 735,000 poses obtained from 249,447 natural compounds.

### Candidate Hits Selection of Docking Results

Starting from the predicted binding poses identified, we analyzed the docking results to identify the most relevant hits (Figure 1). First, we excluded those already reported in the literature as tested against SARS-CoV-2 RdRp activity. This selection process, focused on novelty for this target, yielded 7,958 repurposed and 248,511 natural compounds never reported before as SARS-CoV-2 nsp12 inhibitors. Novel candidate inhibitors were further selected based on significance of the docking scores, retaining only those with prediction scores CSopt at least two standard deviations above the mean (>mean_CSopt_ + 2 SD_CSopt_). The three docking sites were evaluated independently to calculate the average score per site and standard deviation, after which the results were merged.

**Figure 1:**
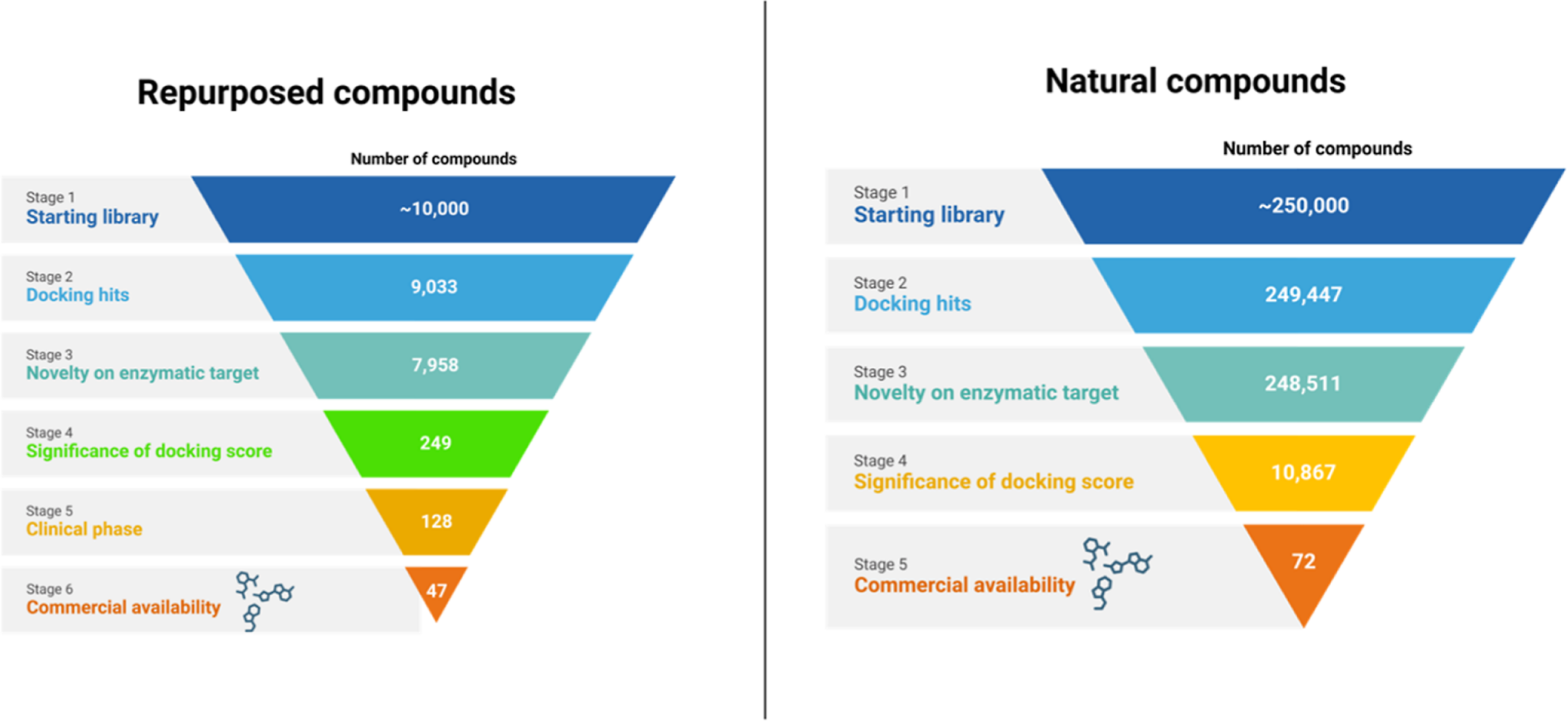
Schematic representation of the selection process of significant docking results from the two libraries of repurposed and natural compounds against SARS-CoV-2 RdRp.

Consequently, 249 repurposed and 10,867 natural-derived molecules were retained. To further refine the selection of potentially safer RdRp candidates, significant docking hits from the repurposed compound library were filtered based on available toxicity data, retaining only those that had at least positively passed phase I in clinical trials (*N* = 128 compounds). This approach could not be applied to natural compounds due to the lack of human toxicity data for the vast majority of molecules in this group. The selection process also considered commercial availability, which led to the selection of a final set of 119 small molecules, composed of 72 natural compounds and 47 repurposed drugs (Figure 1), equally distributed among the ones with a significant docking score, as selected before (data not shown). While some compounds were predicted to bind only to one site, for others, two or three significant docking sites were identified (Tables 1 and S1).

**Table 1:**
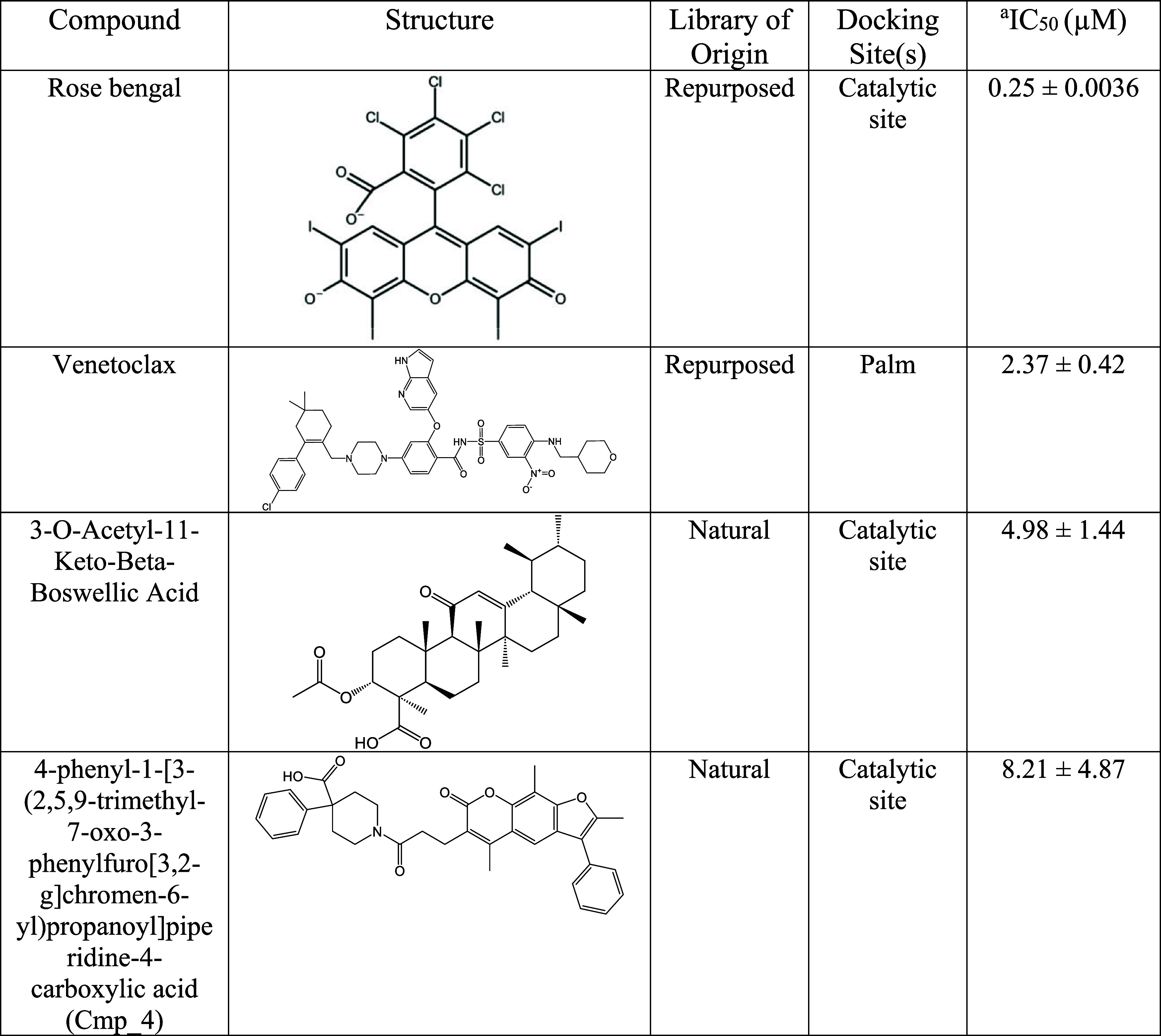
Chemical Structure of the Most Potent Identified RdRp Inhibitor Hits

a Compound concentration required to inhibit by 50% the SARS-CoV-2 RTC enzymatic activity. Data represent the mean and SD of at least 3 independent experiments.

## Establishment of an RTC Assay and Determination of RTC Kinetic Parameters

We first established an enzymatic assay to evaluate the activity of the copurified SARS-CoV-2 RTC complex using a primer-elongation assay on PAGE with a Cy5-labeled 20mer RNA primer and an unlabeled 28mer RNA template. We determined the optimal conditions for the reaction composition such as buffer pH and NaCl and MgCl_2_ concentrations (data not shown). Once the optimal reaction composition was determined, we evaluated the ideal reaction time, which was determined to be 45 min at 37 °C (Figure 2A,B). Subsequently, we determined the optimal enzymatic concentration (Figure 2C,D).

**Figure 2:**
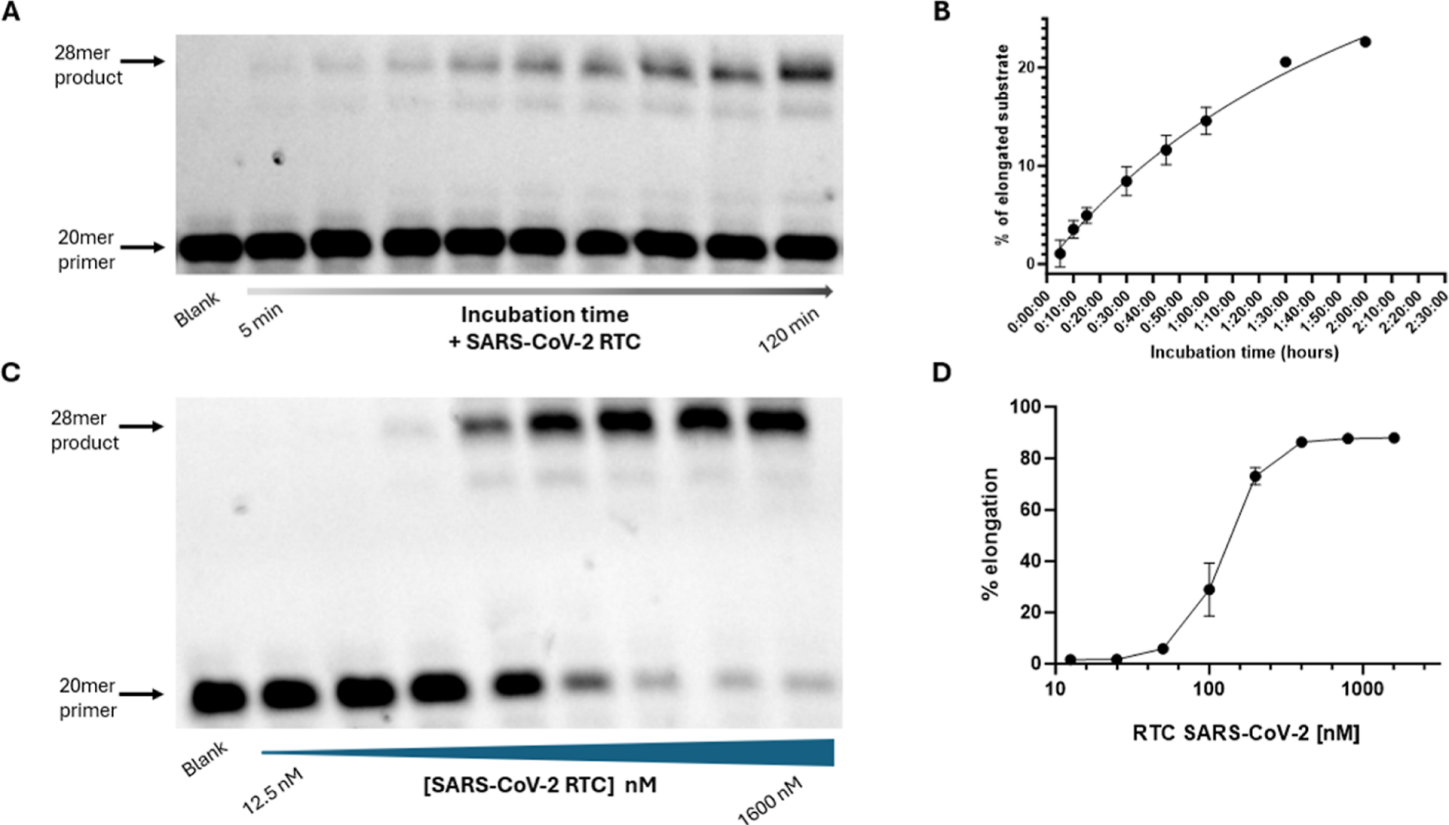
(A) Biochemical characterization of SARS-CoV-2 RdRp activity: Enzymatic activity of the SARS-CoV-2 RTC was measured at various time points (5, 10, 15, 30, 45, 60, 90, and 120 min) using a primer-extension assay resolved on denaturing PAGE; (B) plot of percentage SARS-CoV-2 RTC activity vs elapsed reaction time (from 5 to 120 min, as panel A) as quantified by densitometry from gel in panel A; (C) RdRp activity of different concentrations of SARS-CoV-2 RTC (12.5, 25, 50, 100, 200, 400, 800, and 1600 nM) using a primer-extension assay resolved on denaturing PAGE; (D) plot of the densitometric analysis of gel in panel C to assess the RdRp activity vs different concentrations of SARS-CoV-2 RTC (from 12.5 to 1600 nM) at a time-point of 45 min.

We then calculated the kinetic parameters of both substrates, RNA (Figure 3A) and GTP (Figure 3B), of the first nucleotide incorporated in the T/P RNA duplex used in the assay. Michaelis–Menten plots of initial enzymatic velocity versus substrate concentration resulted in a *K*_M_ of 79.3 nM for RNA and 60.4 nM for GTP.

**Figure 3:**
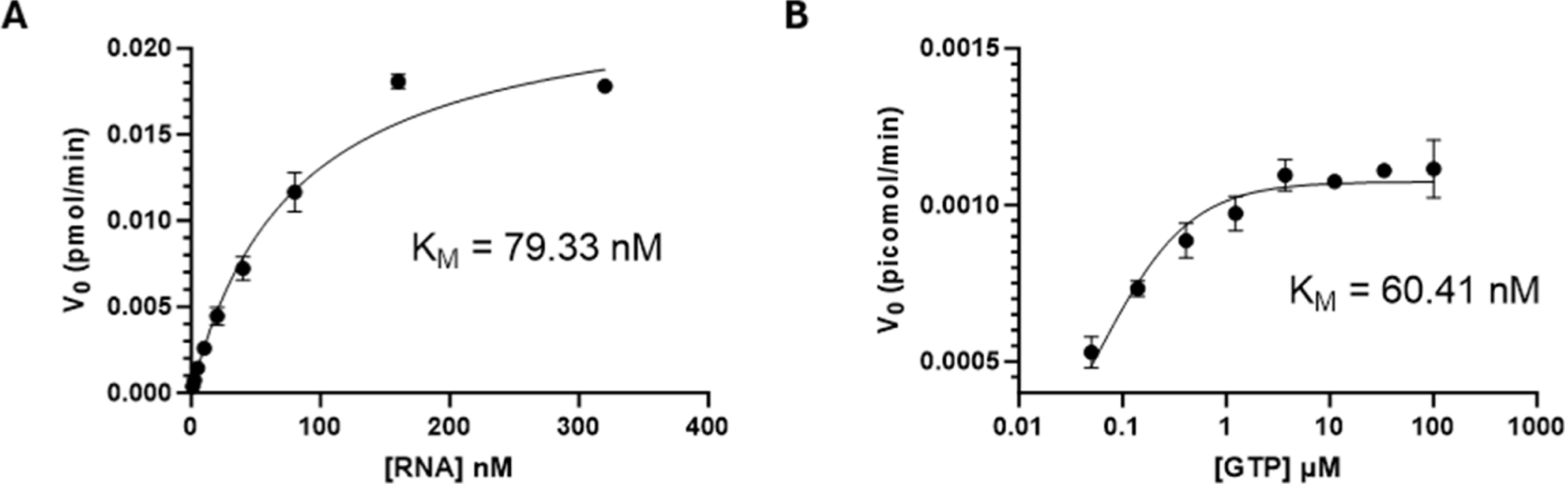
Determination of SARS-CoV-2 RdRp kinetics constants: Michaelis–Menten constant *K*_M_ was determined for SARS-CoV-2 RTC substrates RNA (panel A) and GTP (panel B) by primer-extension assay resolved by denaturing PAGE. Shown graphs are obtained by plotting densitometry data on initial enzymatic velocity vs substrate concentration. *K*_M_ was calculated on GraphPad Prism using the built-in Michaelis–Menten equation.

### Biochemical Hit Confirmation of Selected Candidates

To evaluate the inhibition of the SARS-CoV-2 minimal RTC activity, we exploited the established primer-extension PAGE assay and tested the selected compounds in a primary screen at a single concentration of 100 μM. Simeprevir was used as positive control,^21^ displaying an IC_50_ value of 9.37 ± 3.31 μM. Out of the 119 total hits tested at 100 μM concentration, 42 of them inhibited >50% of the RTC enzyme activity as compared to the DMSO control (35% hit score). Considering compounds based on the library of origin, 38% belonged to the repurposed library (*N* = 16 out of 47–34% hit rate in the library) and 62% to the natural library (*N* = 26 out of 72–36% hit rate in the library). Dose-dependent inhibition curves were obtained for these 42 compounds to calculate their IC_50_ values. Out of these, 13 small molecules were able to inhibit the SARS-CoV-2 RTC with IC_50_ values below 20 μM, and four small molecules showed IC_50_ values below 10 μM (Table 1). Hence, 11% of the tested compounds were identified as potent hits in biochemical assays, clearly demonstrating the strength of the in silico approach. The other compounds that instead showed IC_50_ values between 10 and 100 μM are reported in Table S1. Overall, the most potent compounds were, in order of potency, rose bengal and venetoclax, which belonged to the repurposed library, and 3-acetyl-11-keto-beta-boswellic acid (AKBA) and 4-phenyl-1-[3-(2,5,9-trimethyl-7-oxo-3-phenylfuro­[3,2-g]­chromen-6-yl)­propanoyl]­piperidine-4-carboxylic acid (Cpd_4), which belonged to the natural library. These compounds were further analyzed to assess whether they could have been flagged as potential PAINS (Pan-Assay Interference Compounds), using the SwissADME online tool, where no alert was detected for any of them.

Rose bengal was initially developed as a wool dye, and it has been subsequently used as an ophthalmic diagnostic marker for ocular lesions and laboratory diagnostic tests of brucellosis, and it is also under evaluation as potential treatment against metastatic melanoma and liver tumors.^22–24^ Previous studies have shown that rose bengal was able to inhibit SARS-CoV-2 replication with an EC_50_ value of 0.5 μM,^25^ comparable with the EC_50_ value of 0.18 μM we report, although the molecular target and the mechanistic effects were not identified. In a previous work, rose bengal was actually suggested to be a potential SARS-CoV-2 nsp12 inhibitor, but inhibition potential could not be determined due to compound interference with the dsRNA intercalator used in the RdRp assay.^26^ In fact, we were able to show that rose bengal inhibits the SARS-CoV-2 RTC using a PAGE-based assay. Venetoclax was also reported to be able to inhibit SARS-CoV-2 replication in cell culture with an EC_50_ of 6 μM and it has also been demonstrated to inhibit spike-ACE2 interactions.^27,28^ Hence, the present data suggest that venetoclax might have two independent viral targets, making this a very interesting scaffold hit. The natural compound AKBA derives from *Boswellia serrata* and is a well-known molecule for its anti-inflammatory and immunomodulatory properties, inhibiting COX-1 and leukotriene production, along with TNF-α and IL-1β.^29,30^ Despite no previous direct evidence that AKBA could inhibit SARS-CoV-2 replication, it is worth to mention that it was previously reported that a mixture of three herbal extracts, which included AKBA, had antiviral properties.^31^ Several other works have theoretically proposed AKBA as anti-COVID-19 therapy,^29,30,32,33^ given the profound immune dysregulation induced by the SARS-CoV-2 infection.^34^ Present data hence demonstrate that AKBA has a direct action on viral replication and could be further evaluated for a double targeted therapy. AKBA could potentially act as a direct agent by inhibiting viral replication and could also alleviate viral-induced aberrant immune response by targeting host proteins. To the best of our knowledge, no evidence of antiviral activity was previously reported for Cmp_4 (4-phenyl-1-[3-(2,5,9-trimethyl-7-oxo-3-phenylfuro­[3,2-g]­chromen-6-yl)­propanoyl]­piperidine-4-carboxylic acid).

### Confirming Docking Poses of Most Active Compounds

Based on the top scored docking poses of the in silico screening, it was proposed that venetoclax could bind to the Palm site of nsp12 while the other three most active compounds bind to the catalytic site (Table 1), while none of the most active compounds appeared to dock to the Thumb site. Hence, to confirm this initial result, the binding site of the most active compounds to SARS-CoV-2 RTC was further investigated by more accurate docking simulations. In the case of rose bengal, the best predicted binding pose overlapped with the one of remdesivir monophosphate in PDB 7BV2 (Figure 4A,B), showing that rose bengal makes interactions with amino acid residues Arg555, Arg553, and Lys551, which line the NTP entry channel and play a key role during RNA synthesis. Hence, rose bengal appears to have a binding mode similar to that of remdesivir (Figure 4C), confirming its interaction with the nsp12 active site.

**Figure 4:**
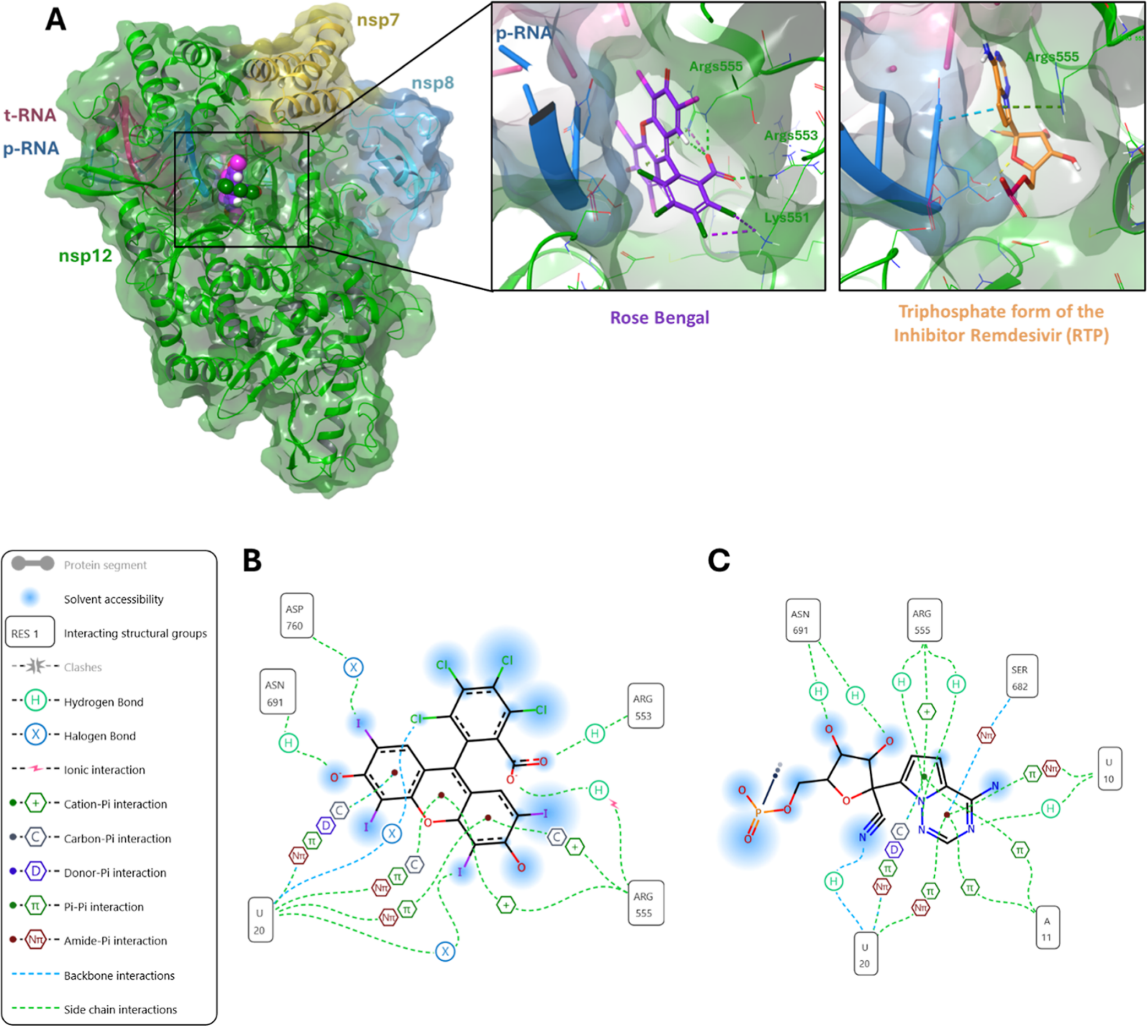
Predicted interaction of rose bengal with SARS-CoV-2 nsp12: (A) Binding pose of rose bengal in the catalytic site of SARS-CoV-2 nsp12 in the presence of RNA. (B) Schematic representation of the predicted interactions of rose bengal with the amino acids of the catalytic site. (C) Schematic representation of the interactions of remdesivir reported in the cryo-EM structure in PDB 7BV2.

Differently, the venetoclax best binding pose predicted an interaction with amino acid residues Arg836 and His439 in an allosteric site that is close to the catalytic active site and that lies in the Palm subdomain and is involved in NTP recognition (Figure 5).

**Figure 5:**
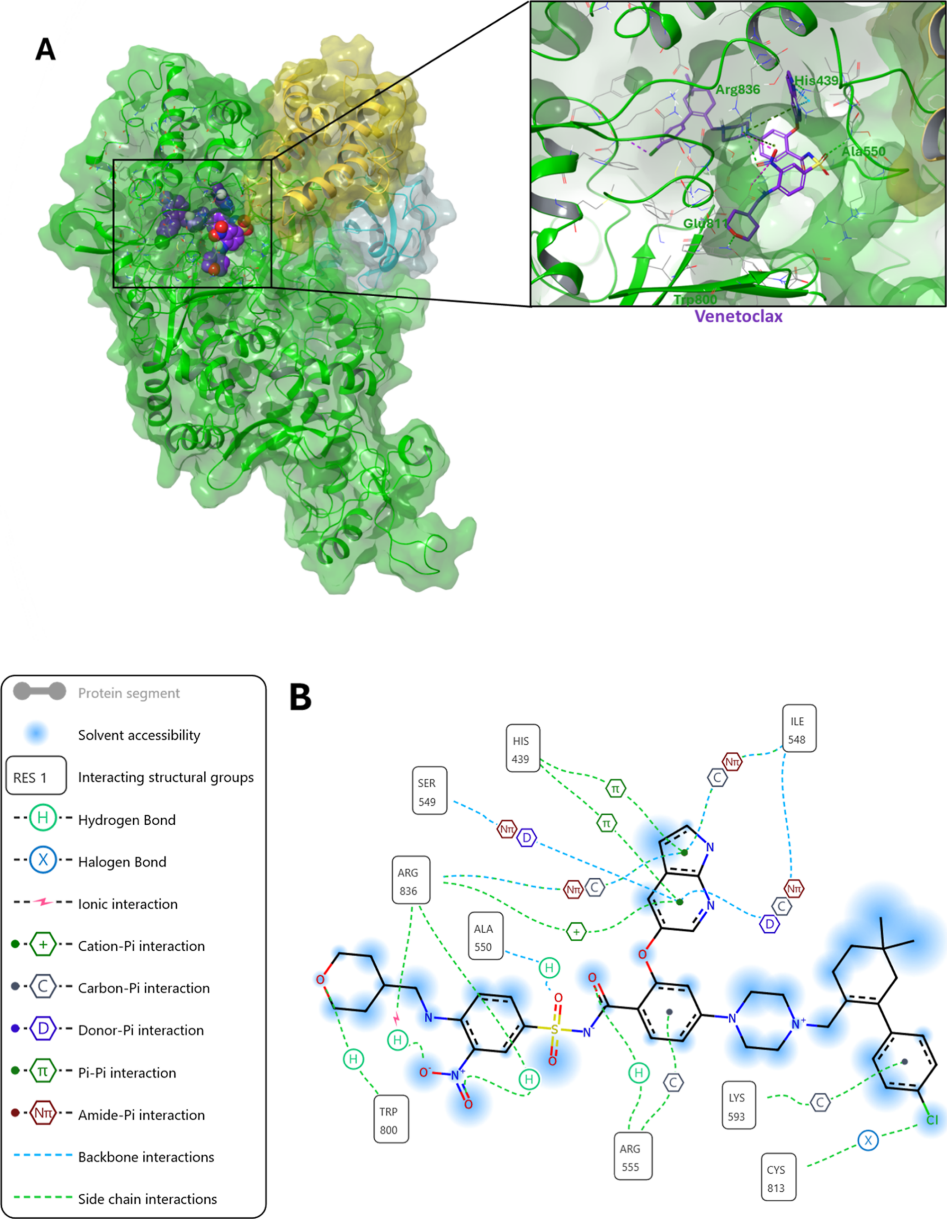
Predicted interaction of venetoclax with SARS-CoV-2 nsp12: (A) Binding pose of venetoclax in the Palm subdomain of SARS-CoV-2 nsp12, which is close to the NTP entry site. (B) Schematic representation of the predicted interactions of venetoclax with the amino acids of the Palm site.

Docking predictions of AKBA and Cmp_4 are reported in Figures S1 and S2.

### Mechanism of Action of Most Potent Hits

In order to further explore the potential of the two most potent hits, rose bengal and venetoclax were better characterized in their interaction with SARS-CoV-2 RTC. Our in silico modeling predicted that rose bengal docks in the catalytic site of nsp12 in the presence of the RNA T/P duplex, forming key interactions with amino acids Arg555, Arg553, and Lys551, which are located near the NTP entry channel. Differently, venetoclax was predicted to bind to an allosteric site in the Palm subdomain, which is also close to the NTP entry channel. Given their predicted divergent mechanisms of interaction with nsp12, we further investigated it by competition assays with both RdRp substrates, the RNA template and a nucleotide. In particular, GTP was chosen as the representative nucleotide for these studies as it was the first nucleotide to be incorporated in our elongated primer, simplifying the densitometric analyses, which could be complicated by incomplete stalled products in the presence of all four nucleotides. Results showed that rose bengal, when competing either against RNA or GTP RTC substrates, caused a decrease in both apparent *V*_max_ and *K*_M_ of SARS-CoV-2 RdRp in Michaelis–Menten plots (Figure 6A,C). Data analysis with Lineweaver–Burk plots showed that in both cases, the lines intersect below the negative half of the *X*-axis, which is indicative of a mixed model of inhibition (Figure 6B,D).

**Figure 6:**
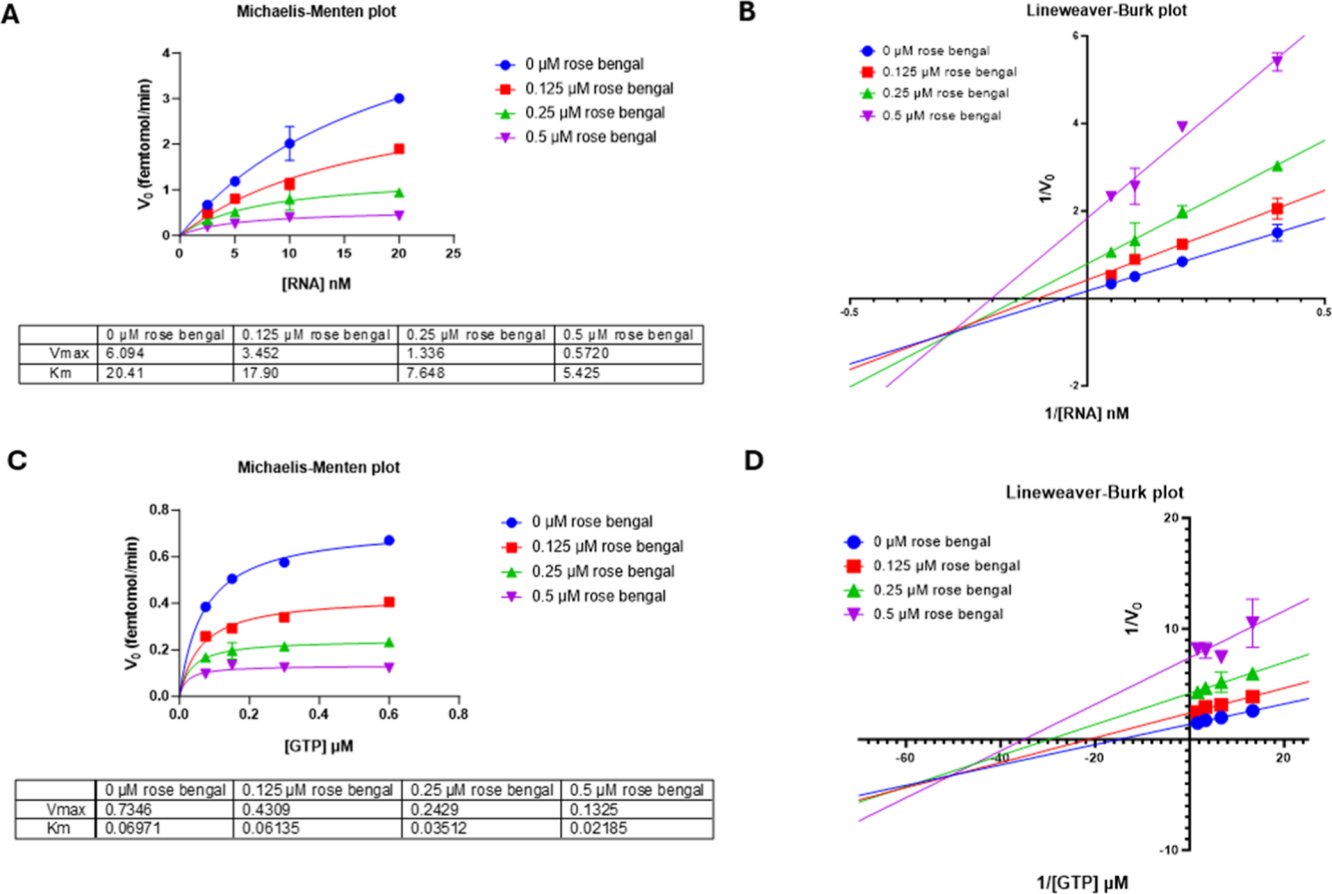
Kinetics of SARS-CoV-2 RTC enzyme activity inhibition by rose bengal: (A) Michaelis–Menten constant apparent *K*_M_ and *V*_max_ parameters for rose bengal were assessed by plotting initial velocity of SARS-CoV-2 RTC in the presence of different concentrations of compounds vs different concentrations of substrate RNA and (B) Lineweaver–Burk plot of reciprocal initial velocity vs reciprocal substrate concentration. (C) Similarly, different concentrations of GTP yielded the Michaelis–Menten constant apparent *K*_M_ and *V*_max_ parameters and (D) Lineweaver–Burk plot of reciprocal initial velocity vs reciprocal substrate concentration.

Since DNA-binding properties for rose bengal have been reported before,^35^ which could interfere with the observed mechanism of action, we performed Microscale Thermophoresis/Spectral Shift binding assays using Monolith X (NanoTemper), which revealed no evidence of interaction between rose bengal and our RNA substrate (data not shown).

Differently, results showed that venetoclax, when competing either against RNA or GTP RTC substrates, determined a decrease in apparent *V*_max_ while *K*_M_ was unaffected (Figure 7A,C). Data analysis with Lineweaver–Burk plots showed that the line intersection reveals a noncompetitive mode of inhibition (Figure 7B,D). To rule out nonspecific enzyme degradation by venetoclax, we performed a binding check at a single concentration of 500 μM of compound using Microscale Thermophoresis/Spectral Shift with the Monolith X instrument (NanoTemper). The results demonstrated specific binding of venetoclax to the SARS-CoV-2 minimal RTC at this concentration, thereby supporting the specific inhibitory activity of the compound (Figure S3).

**Figure 7:**
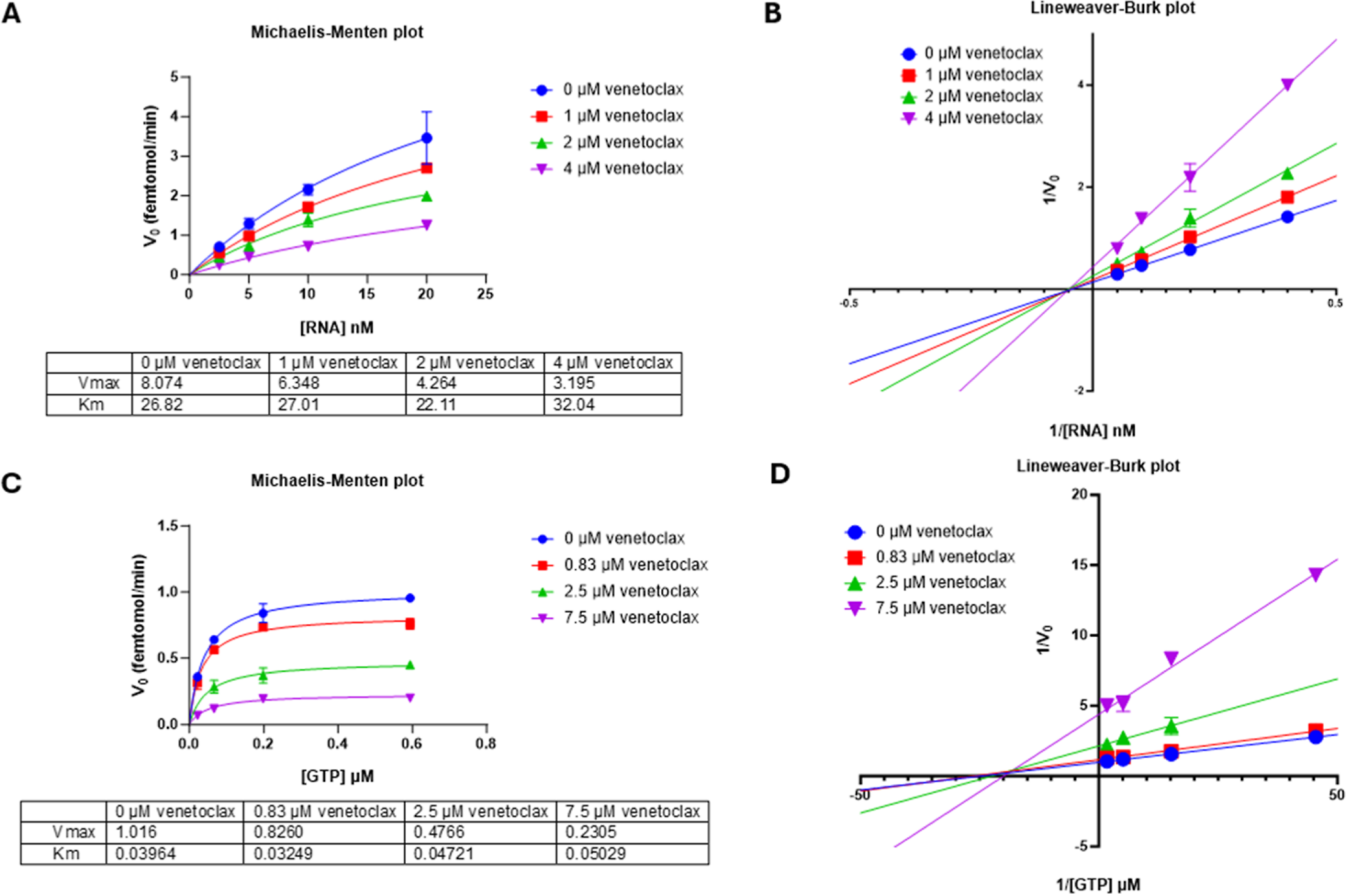
Kinetics of SARS-CoV-2 RTC enzyme activity inhibition by venetoclax: (A) Michaelis–Menten constant apparent *K*_M_ and *V*_max_ parameters for venetoclax were assessed by plotting initial velocity of SARS-CoV-2 RTC in the presence of different concentrations of compounds vs different concentrations of substrate RNA and (B) Lineweaver–Burk plot of reciprocal initial velocity vs reciprocal substrate concentration. (C) Similarly, different concentrations of GTP yielded the Michaelis–Menten constant apparent *K*_M_ and *V*_max_ parameters and (D) Lineweaver–Burk plot of reciprocal initial velocity vs reciprocal substrate concentration.

These results align with the docking predictions, as rose bengal yielded a significant docking score for the catalytic site of SARS-CoV-2 nsp12, while venetoclax docked in its Palm site. Interestingly, the evaluation of the inhibition kinetics supported a mixed model of inhibition for rose bengal and a noncompetitive model of inhibition for venetoclax. Hence, the biochemical evaluations appeared to confirm the results of the docking predictions, with the two compounds interacting with the enzyme at different binding sites. In fact, the close proximity, but not overlap, of the rose bengal binding site with both RNA and NTP binding sites may well justify the mixed model mechanism of inhibition, which suggests that rose bengal modulates the interactions between the enzyme and its substrates. In addition, venetoclax binding to the allosteric Palm subdomain is fully supported by our biochemical results.

### Molecular Dynamics Simulations of Most Potent Hits

Molecular dynamics (MD) simulations were conducted on the complexes formed between the compounds listed in Table 1 and the protein target. Each compound underwent 250 ns of simulation to evaluate the behavior of each ligand and to identify possible effects on the target’s conformation. As shown in Figure 8, while the overall impact on the protein was comparable across all cases (protein RMSD did not exceed 2.5 Å), analysis of the ligands’ RMSD revealed interesting trends. Notably, the most potent compounds, rose bengal and AKBA, exhibited extremely low ligand RMSD values, supporting the conclusion that these compounds adopted particularly stable and efficient binding modes. In contrast, Cmp_4, which demonstrated lower potency, showed greater ligand fluctuations, indicating that additional conformational searching was required to achieve an optimal binding orientation.

**Figure 8:**
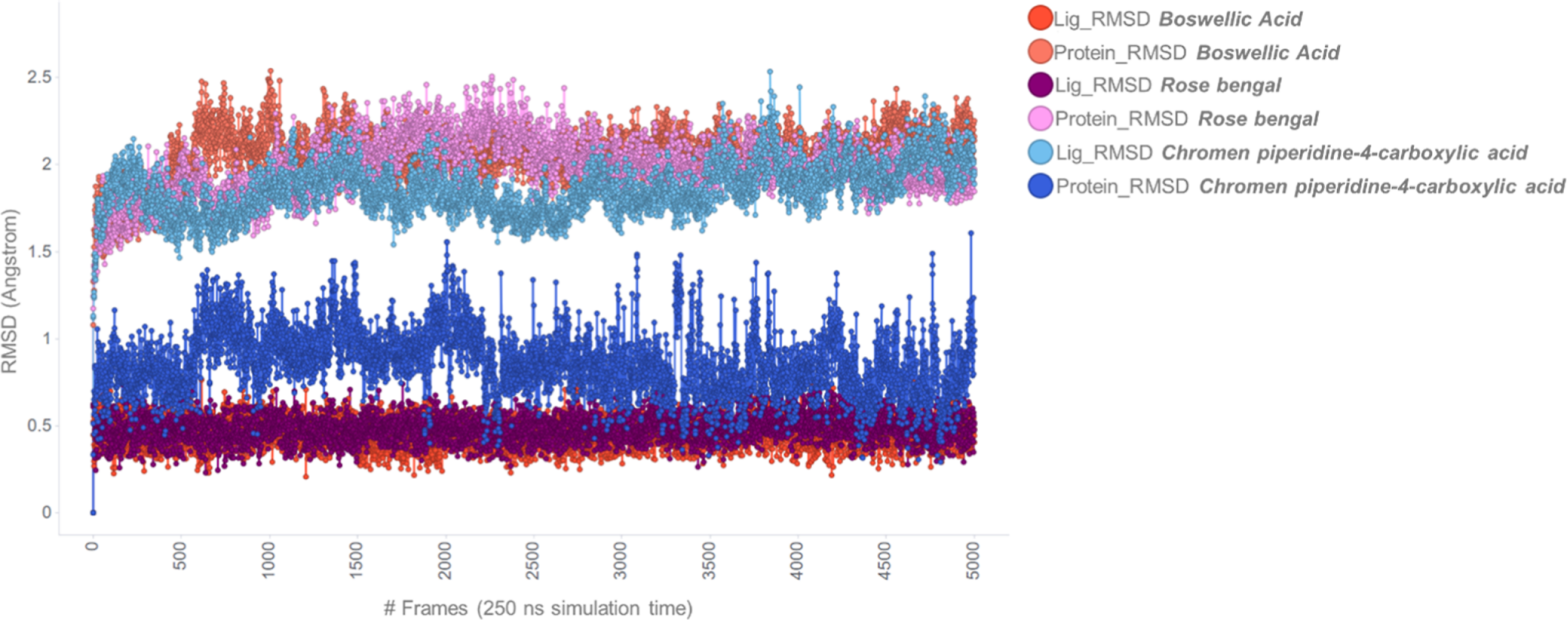
MD simulation analysis of catalytic site binders. The following plots show the progression of the MD simulations for the three catalytic site ligands. The left *Y*-axis shows protein RMSD changes over time (*X*-axis). Protein frames are aligned to a reference backbone, and then RMSD is calculated based on selected atoms. RMSD monitoring reveals structural changes during the simulation; shifts of 1–3 Å are normal for small globular proteins, while larger deviations suggest major conformational change.

The analysis was also performed by comparing the two most potent compounds, rose bengal and venetoclax. Figure 9 shows the behavior of venetoclax in comparison with rose bengal. It is important to note that these two compounds bind to different regions of the protein and, as a result, their effects are entirely distinct. Apparently, venetoclax exerts its activity, demonstrating excellent potency, by inducing a significant conformational change within the first 1,500 frames (corresponding to the initial 100 ns). Subsequently, both the ligand and protein conformations remain stable throughout the end of the simulation, as indicated by the steady RMSD values for both the ligand and protein.

**Figure 9:**
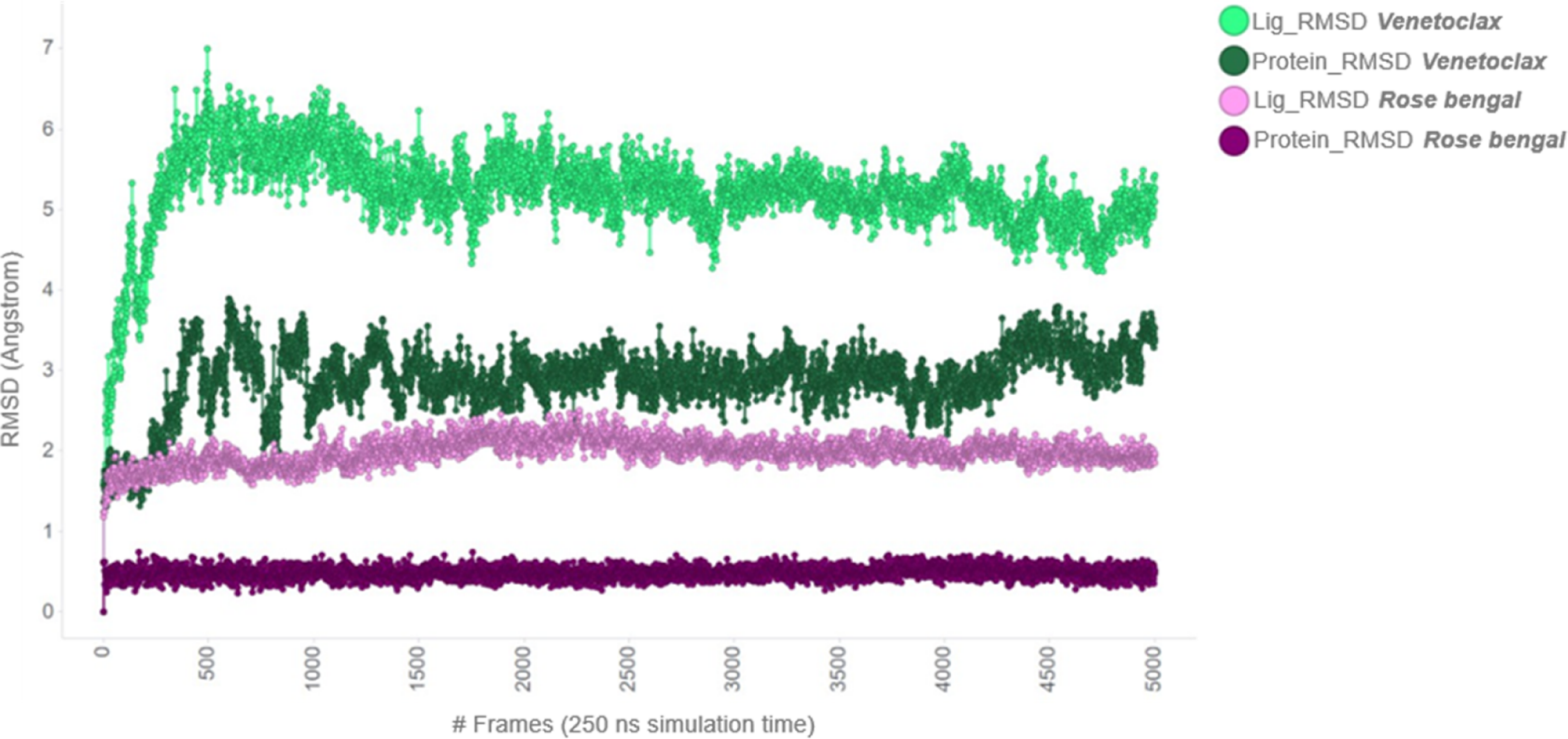
MD simulation analysis of venetoclax in comparison to rose bengal. The following plots show the progression of the MD simulations for venetoclax in the allosteric pocket, in comparison with data obtained for rose bengal in the catalytic site. The left *Y*-axis shows protein RMSD changes over time (*X*-axis).

### Inhibition of SARS-CoV-2 Replication by the Most Potent Hits

Once we confirmed that the in silico screening led to the identification of compounds that could effectively inhibit the SARS-CoV-2 RTC activity in biochemical assays binding, as predicted, to the catalytic sites or to the Palm site, we wanted to verify whether these compounds were also able to inhibit viral replication. Hence, the four most potent compounds on the SARS-CoV-2 RTC activity were then evaluated against SARS-CoV-2 replication in vitro using compound GC376 as positive control.^36,37^ While none of the tested compounds displayed any toxicity in the cell system at concentrations up to 100 μM, they potently inhibited SARS-CoV-2 replication in the nanomolar or low micromolar range with a high SI, at drug concentrations comparable to the IC_50_ values in the biochemical assay (Table 2).

**Table 2:** Antiviral Effect of Selected Compounds

compound	EC_50_ (μM)^a^	CC_50_ (μM)^b^	SI^c^
rose bengal	0.18 ± 0.02	>100	>546.5
venetoclax	0.85 ± 0.08	>100	>117.9
AKBA	4.81 ± 2.15	>100	>20.8
Cpd_4	2.61 ± 0.18	>100	>38.36
GC376	0.06 ± 0.03	>100	>5,882

a Compound concentration required to inhibit SARS-CoV-2 replication by 50%. Data represent the mean and SD of at least 3 independent experiments.

b Compound concentration required to reduce Vero E6 GFP viability by 50%. Data represent the mean and SD of at least 3 independent experiments.

c Selective index: ratio of CC_50_/EC_50_.

### In Silico ADMET Analysis of Top-Ranked Compounds

A subset of key ADMET-related descriptors was selected from the SwissADME output to highlight the most relevant pharmacokinetic and druglike properties of the compounds (Table 3).

**Table 3:** ADMET Analysis of Selected Compounds

compound	MW (Da)	TPSA	Consensus log P	ESOL log S	GI absorption	BBB permeant	*P*-gp substrate	CYP3A4 inhibitor	Lipinski violations	Synthetic accessibility
rose bengal	1049.85	93.4	2.78	–11.42	low	no	yes	no	2	3.72
venetoclax	868.44	183.09	6.12	–9.78	low	no	yes	no	2	6.05
3-*O*-acetyl-11-keto-beta-boswellic Acid (AKBA)	512.72	80.67	5.74	–7.37	low	no	yes	no	2	6.41
4-phenyl-1-[3-(2,5,9-trimethyl-7-oxo-3-phenylfuro[3,2-g]chromen-6-yl)propanoyl]piperidine-4-carboxylic acid (Cmp_4)	563.64	100.96	5.63	–6.98	low	no	no	no	1	4.65

As shown in Table 3, all compounds violate at least one of Lipinski’s rules, primarily due to high molecular weight and/or lipophilicity. Rose bengal and venetoclax exhibit particularly high molecular weights (>800 Da), while AKBA and Cmp_4 are closer to the oral space threshold (MW ≈500–560 Da).

All compounds showed low predicted gastrointestinal absorption, which may limit their oral bioavailability. The BOILED-Egg plot based on SwissADME predictions is provided in the Supporting Information (Figure S4). None are predicted to permeate the blood–brain barrier. Rose bengal and AKBA are substrates of *P*-glycoprotein, potentially reducing intracellular availability due to efflux.

Notably, none of the compounds are predicted to inhibit CYP3A4, reducing the concern for metabolic liabilities. Solubility predictions (ESOL LogS) ranged from −11.4 to −6.9, indicating low solubility across the board, though Cmp_4 performs comparatively better.

Overall, Cmp_4 displays the most balanced profile among the candidates, with only 1 Lipinski violation, no *P*-gp interaction, and a moderate synthetic accessibility score.

## Conclusions

The threat that SARS-CoV-2 still poses to global health strengthens the need for effective and safe drugs to fight emerging viral infections. Due to climate change and anthropization of wild environments, the risk of increase in frequency and severity of zoonotic spillovers appears to be more and more concrete in the near future.^38,39^ In this context, SARS-related coronaviruses represent a significant threat as more than 66,000 spillover events/year are already estimated to occur in Southeast Asia, suggesting that bat-to-human spillover is more common than expected.^40^ Considering that the coronavirus RdRp is highly conserved over time^41,42^ and among other (+)­ssRNA viruses,^43^ we aimed to target the SARS-CoV-2 RTC to identify novel antiviral agents. The in silico approach allowed us to screen >250,000 compounds, from two libraries, that could interact with either the active site or/and two conserved allosteric sites in the Palm and Thumb subdomains and to select 119 small molecules, identifying four small molecules able to inhibit SARS-CoV-2 RdRp activity with an IC_50_ below 10 μM. We also showed that these four most potent compounds also inhibited viral replication in cell-based assays at comparable drug concentrations, further demonstrating the efficacy of our in silico approach. Docking results were further validated by biochemical competition assays, with inhibitory mechanisms of action in line with the docking prediction and MD simulation of the two most potent compounds.

In conclusion, we report a computer-aided drug discovery approach (CADD) for the in silico screening of around 250000 compounds leading to the identification of 42 active compounds against the RdRp of SARS-CoV-2, among which four showed IC_50_ and EC_50_ values in the nanomolar or low micromolar range. The approach allows for a potentially accelerated development of promising compounds with an expanded chemical space that remains feasibly explorable only within these platforms. These hits, given the structural conservation among viral RdRp, represent four new scaffolds with broad-spectrum antiviral potential. While this study provides valuable insights toward the development of effective treatments against emerging variants and future coronaviruses, it is limited by the lack of experimental validation of the target site of the compounds, which can be confirmed by site-directed mutagenesis and/or cryo-EM structure of the ligand–protein complexes, and the lack of in vivo efficacy data of the identified compounds. Additionally, in silico ADMET analysis suggested that improvement in the drug-likeness of the compounds is further required. Future research on these missing points may provide comprehensive data for the future development of the identified compounds as antiviral drugs.

## Materials and Methods

### Library and Protein Structure Preparation

A protocol was applied to assemble and curate data related to marketed drugs and compounds in clinical phases as well as withdrawn and discontinued ones from multiple sources, including the Clarivate’s Cortellis Drug Discovery Intelligence (CCDI) database, DrugBank, and DrugMap. The subset of marketed drugs, compounds in clinical phases (I, II, and III), and withdrawn and discontinued compounds is hereafter referred to as “Safe in Man” (SIM), containing ∼11,000 compounds.^44^ Virtual screening studies were performed on a repurposed compound library, containing a unique list of about 10,000 drugs, which comprise the set of Safe-In-Man drugs, commercialized or under active development in clinical phases and retrieved from the Integrity database (https://clarivate.com/cortellis/solutions/pre-clinical-intelligence-analytics/), and a natural deriving compound library, containing a total of about 250,000 molecules. All compounds were converted to 3D structures and prepared by using Schrödinger’s LigPrep tool. This process generated multiple states for stereoisomers, tautomers, ring conformations (one stable ring conformer by default), and protonation states. In particular, another Schrödinger package, Epik, was used to assign tautomers and protonation states that would be dominant in a selected pH range (pH = 7 ± 1). Ambiguous chiral centers were enumerated, allowing a maximum of 32 isomers to be produced from each input structure. Then, energy minimization was performed with the OPLS3 force field. The protein was prepared using the Maestro Protein Preparation Wizard. Hydrogen atoms were added, and water molecules were removed from the protein structure.

### Docking Engine

The docking simulations were performed by using LiGen. LiGen, proprietary software developed by Dompé Farmaceutici SpA, implements a geomrigid fitting procedure combined with rigid body minimization. Specifically, the docking engine follows a specific workflow during which three docking scores are computed: first, the Pacman Score (PS) estimates a geometric fitting by evaluating the interaction between a ligand pose and the pocket based on shape and volume complementarity. Then, the Chemical Score (CS), which encodes for the ligand binding interaction energy, is calculated by an in-house-developed scoring function. The last step involves a rigid body minimization of the docked ligand within the binding site, at the end of which a third score called the optimized chemical score (CSopt) is evaluated. All poses that do not fulfill geometric fitting or threshold values of user-defined specific parameters are discarded. The GENEOnet tool was utilized to define the protein binding pockets to guide the docking experiments.^45^ This proprietary software integrates the geometric and explainability features of GENEOs with a network architecture, forming a novel knowledge-based machine learning paradigm. GENEOnet leverages knowledge such as lipophilicity, hydrophilicity, and electrostatic information, which are essential for identifying binding sites. For each chemical–physical parameter, a GENEO^46^ is defined to identify areas with optimal values for these parameters. Molecules were prioritized according to the score value (CSopt), which predicts the binding affinity of the molecules in the protein binding site. Samson software, integrated to LiGen as a graphical interface, was used to generate the ligand interaction diagrams shown in Figures 4B and 5B.

### Selection of Candidate Hits

Data from docking predictions were analyzed with PipelinePilot (BIOVIA), which implemented in-house protocols. We cross-referenced the names of each compound against Cortellis Drug Discovery Intelligence (CDDI, Clarivate), EMBASE (Elsevier), and NCATS/NIH Covid19 HTS (https://opendata.ncats.nih.gov/covid19) databases by using in-house PipelinePilot protocols, to filter out the compounds that had not been previously reported as tested against SARS-CoV-2 RdRp activity. We selected the hits with a CSopt score higher than the mean value per site, plus two standard deviations. Repurposed compounds were further filtered based on the clinical phase using data from https://clinicaltrials.gov/. Most potent compounds against enzymatic RdRp function of SARS-CoV-2 were assessed for their potential PAINS-like behavior (Pan-assay interference compounds) and ADMET analysis using the SwissADME online tool.^47^

### SARS-CoV-2 nsp12/7/8 Copurification

The SARS-CoV-2 nsp12/7/8 (RTC) complex was coexpressed in the *E. coli* BL21-Gold (DE3) strain using plasmid pRSFDuet-1­(nsp8 nsp7)­(nsp12) (Addgene #165451), following a previously reported protocol,^48^ with minimal modifications. Briefly, recombinant nsp12, nsp7, and nsp8 were coexpressed in bacteria grown in LB medium with 0.05 mM IPTG at 20 °C for 18 h in 135 rpm agitation. After centrifugation, bacterial pellets were resuspended in a buffer containing 50 mM Tris-HCl at pH 8.0, 500 mM NaCl, and 10 mM imidazole, supplemented with EDTA-free protease inhibitor cocktail (cOmplete Mini, Roche). Cells were lysed by ultrasonication, and the lysate was centrifuged at 16,000 rpm and 4 °C for 45 min. The resulting supernatant was applied to a HisTrap HP 5 mL column (Cytiva). The column was washed with 25 mL of lysis buffer, and the protein was eluted using a gradient of elution buffer (50 mM Tris-HCl pH 8.0, 500 mM NaCl, and 500 mM imidazole). The presence of proteins in selected fractions was confirmed by SDS-PAGE, after which fractions were diluted 10-fold with 50 mM Tris-HCl pH 8.0 and loaded onto a HiTrap Q HP 5 mL column (Cytiva). Proteins were eluted with a gradient of elute buffer (50 mM Tris-HCl at pH 8.0 and 1 M NaCl). Selected fractions after SDS-PAGE were pooled, concentrated, and further purified by size-exclusion chromatography (HiLoad 16/600 Superdex 200 pg column, Cytiva) in a buffer containing 50 mM Tris-HCl pH 8.0, 300 mM NaCl, and 1 mM MgCl_2_. Protein purity was confirmed by SDS-PAGE, after which fractions were pooled, concentrated at ≈2 mg/mL, flash-frozen in liquid nitrogen, and stored at −80 °C.

### SARS-CoV-2 RdRp Enzymatic Assay

The RdRp activity of the SARS-CoV-2 nsp12 and nsp7/8 complex was assessed by a primer-extension assay on denaturing urea-PAGE, similarly to a previously reported protocol.^49^ Briefly, an RNA template (28 nt, 3′-UCUUGGACAACUUGUUUUCGCGUACGAU-5′) was annealed with a Cy5-labeled RNA primer (20 nt, Cy5–5′-AGAACCUGUUGAACAAAAGC-3′) in a 1:1 ratio in 50 mM Tris-HCl pH 8.0 and 150 mM NaCl at a final concentration of 10 μM. The annealing mixture was denatured at 95 °C for 10 min and then gradually cooled to 4 °C overnight. SARS-CoV-2 RTC enzyme kinetics was determined by preincubating 50 nM of SARS-CoV-2 RTC in 5% DMSO at 37 °C for 30 min in reaction buffer (20 mM HEPES pH 8.0, 25 mM NaCl, 1 mM MgCl_2_, 10 mM DTT, 0.01% Tween 20, 5 nM RNA template, RNase inhibitor [Euroclone]). Reactions were initiated by adding 20 μM rNTPs, followed by incubation at 37 °C. Then, 40 μL of stopping buffer (formamide with 4% EDTA) was added to each reaction at different time-points. The reactions were denatured at 95 °C for 10 min and resolved by 15% urea-PAGE (7 M urea, 19:1 acrylamide/bis-acrylamide). Gels were scanned using a ChemiDoc Imager (Bio-Rad), and images were analyzed via densitometry using Image Lab 4.0 to quantify elongated vs nonelongated primer bands. The curve of enzymatic activity over the reaction time was generated with GraphPad Prism 10.1.

Optimal nonsaturating enzyme concentration was determined in the conditions reported above. Briefly, 2-fold serially diluted SARS-CoV-2 RTC was preincubated in 5% DMSO for 30 min, and enzymatic activity was assessed by blocking reactions after 45 min. Reactions were resolved by urea-PAGE as above, and data was analyzed with GraphPad 10.1.

### SARS-CoV-2 RdRp Enzymatic Inhibition Assay

For the evaluation of the compounds’ efficacy on the RdRp, the copurified SARS-CoV-2 RTC complex was preincubated at a final concentration of 150 nM in 5% DMSO in the presence of serially diluted compounds at 37 °C for 30 min in reaction buffer. Inhibition assay was conducted as reported above. Eight points dose–response curves were generated with GraphPad Prism 10.1 by fitting the log_10_ inhibitor concentration against the normalized response using a variable-slope, nonlinear regression. Simeprevir was used as internal positive control.^21^

### SARS-CoV-2 RdRp Competition Assay

The Michaelis–Menten constant (*K*_M_) of GTP and RNA substrates was assessed in the assay conditions reported above. Initial velocity was calculated by dividing the enzymatic activity by the reaction time for each reaction. Initial velocity vs substrate concentration was plotted using GraphPad 10.1 with a nonlinear regression using the Michaelis–Menten equation for apparent *K*_M_ determination.^50^ The Lineweaver–Burk plot was generated by calculating and plotting with linear regression on GraphPad Prism 10.1 the reciprocal of the initial velocities and substrate concentrations.^51^

### Molecular Dynamics Simulations

The molecular dynamics (MD) simulations were performed using the Desmond Multisim protocol.^52^ Initially, the system was prepared by solvating it in an orthorhombic box with a 10 Å buffer of TIP3 (transferable intermolecular potential 3-point) water molecules. Counter ions were added to neutralize the net charge of the system. During the early phase, the Multisim method enabled structural equilibration and relaxation, ensuring a well-matured simulation environment. The simulations were run at a constant pressure of 1 atm and a temperature of 310 K. Both thermostatting and barostatting employed the Martyna–Tobias–Klein method with coupling constants of 0.5 ps for the thermostat and 2.0 ps for the barostat. To enhance computational efficiency, all hydrogen atom positions were constrained using the M-SHAKE algorithm, permitting a time step of 2 fs. Long-range electrostatic interactions were evaluated at each time step using the Particle Mesh Ewald (PME) method, with a cutoff radius set at 10 Å. For each system, 250 ns of simulation were carried out.

### Evaluation of Compounds Cytotoxicity in the Vero E6 Cell Line

As previously described,^53^ stably transfected Vero E6 expressing GFP (Janssen Pharmaceutical) were seeded in a black 96-well plate (ThermoFisher) at a density of 10^4^ cells/well in DMEM supplemented with 10% heat-inactivated FBS (Gibco), 1% penicillin/streptomycin (Euroclone), and 0.075% Na-bicarbonate and incubated at 37 °C in 5% CO_2_. The following day, the cells were treated with serially diluted compounds in a culture medium in the presence of 2 μM *P*-gp inhibitor CP-100356. After 24 h at 37 °C, the culture medium was removed and fluorescence at 485/535 nm, for exc/em wavelength, respectively, was read with plate reader Victor Nivo (PerkinElmer). Dose–response curves of cell viability were generated with GraphPad Prism 10.1 by fitting the log_10_ compound concentration against the normalized fluorescence of treated cell viability vs nontreated controls using a variable-slope, nonlinear regression.

### Evaluation of Compounds in SARS-CoV-2 Plaque Assay

Vero E6 cells were seeded in 96-well plates, as reported above for Vero E6 GFP cells. After 24 h of incubation, the medium was removed and cells were infected with SARS-CoV-2 (BetaCov/Belgium/GHB-03021/2020 provided by KU Leuven) at a MOI of 0.01, in the presence of serial dilutions of compounds in the culture medium supplemented with 2 μM *P*-gp inhibitor CP-100356. Viral inoculum was removed after 1.5 h at 37 °C and replaced with a medium supplemented with compounds and CP-100356. 24 h postinfection (hpi), viral load in supernatants of mock-infected, nontreated infected, and treated wells was titrated by plaque assay in 24-well plates, previously seeded with Vero E6 at 1.5 × 10^6^ cell/mL (400 μL/well). After 1.5 h at 37 °C to allow viral adsorption, cell monolayers were overlaid with 400 μL of 1% methylcellulose solubilized in DMEM supplemented with 10% heat-inactivated FBS, 1% penicillin/streptomycin, and 0.075% Na-bicarbonate. 72 hpi, the overlaying medium was removed, and cells were washed with PBS and fixed with 300 μL of 4% paraformaldehyde for 2 h, after which cell monolayers were stained with 1% crystal violet in 10% EtOH for 15 min. EC_50_ was calculated by normalization of plaque counts of treated wells to PFUs in nontreated controls. Data was analyzed with GraphPad 10.1. Compound GC376 was used as internal positive control.^54^

## Supplementary Material



## Notes

Chemical structure of the identified RdRp inhibitor hits, predicted interaction of AKBA with SARS-CoV-2 nsp12, predicted interaction of Cmp_4 with SARS-CoV-2 nsp12, binding check of SARS-CoV-2 RTC and venetoclax, and BOILED-Egg plot of top-ranked compounds (PDF)
