# Supplementary material for: Dual-Site Inhibition of SARS-CoV‑2 RNA-Dependent RNA Polymerase by Small Molecules Able to Block Viral Replication Identified through a Computer-Aided Drug Discovery Approach
Source: ACS Infect Dis. 2025 Sep 26;11(10):2821–35. doi: 10.1021/acsinfecdis.5c00517 (PMC12519464; doi:10.1021/acsinfecdis.5c00517)
Supplement: Supplementary file 1 [file id5c00517_si_001.pdf]

SUPPORTING INFORMATION

Dual-Site Inhibition of SARS-CoV-2 RNA-Dependent RNA Polymerase By Small Molecules Able to Block Viral Replication Identified Through a Computer-Aided Drug Discovery Approach

Paolo Malune[1], Daniela Iaconis[2], Candida Manelfi[2], Stefano Giunta[1], Roberta Emmolo[1], Filippo Lunghini[2], Annalaura Paulis[1], Carmine Talarico[2], Angela Corona[1], Andrea Rosario Beccari[2], Enzo Tramontano[1], Francesca Esposito[1]*.

[1]Department of Life and Environmental Sciences, University of Cagliari, Cittadella Universitaria di Monserrato, 09124 Cagliari, Italy.

[2]EXSCALATE, Dompé farmaceutici S.p.A., Via Tommaso De Amicis, 95, Napoli, 80131, Italy.

*Corresponding author: francescaesposito@unica.it

Table S1: Chemical structure of the identified RdRp inhibitor hits

Compound	Structure	Library of Origin	Docking Site(s)	$^{a}IC_{50}$ (μM)
3-Epiursolic Acid	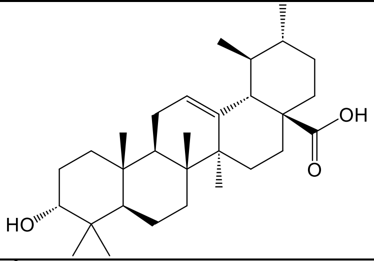	Natural	Catalytic Palm Thumb	11.59 ± 3.97
3-[[4-[(1S)-1-[3-(3,5-dichlorophenyl)-5-(6-methoxynaphthalen-2-yl)pyrazol-1-yl]ethyl]benzoyl]amino]propanoic acid	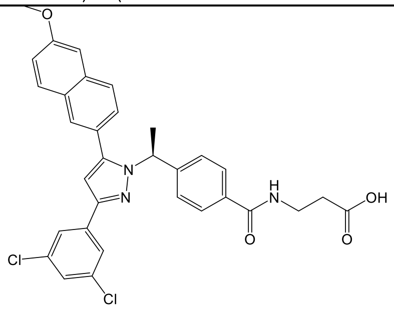	Repurposed	Catalytic	11.82 ± 6.43
Omaveloxolone	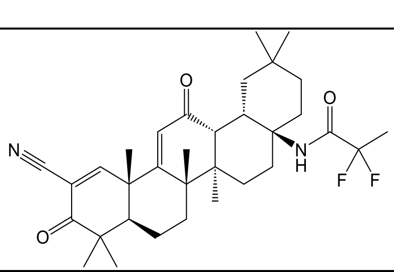	Repurposed	Catalytic Palm Thumb	13.02 ± 4.28

Dryocrassin ABBA	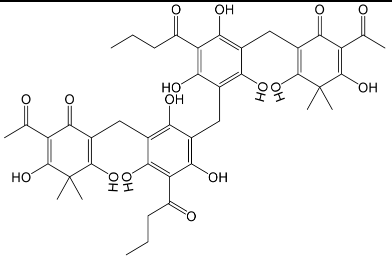	Natural	Catalytic Palm	14.5 ± 5.46
Meclinertant	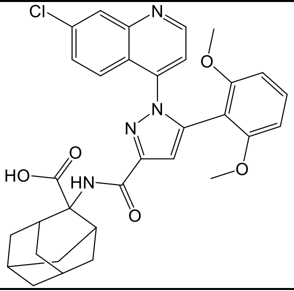	Repurposed	Catalytic Palm Thumb	15.45 ± 2.77
3-methyl-5-propan-2-yl-8-(pyridin-4-ylmethyliminomethyl)-2-[1,6,7-trihydroxy-3-methyl-5-propan-2-yl-8-(pyridin-4-ylmethyliminomethyl)naphthalen-2-yl]naphthalene-1,6,7-triol	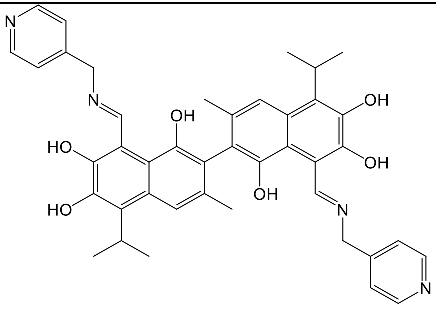	Natural	Catalytic Palm	16.05 ± 0.32
Narcissiflorine	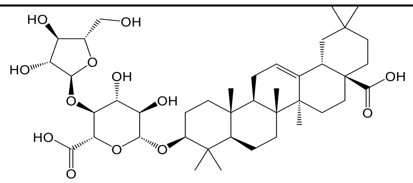	Natural	Catalytic	17.24 ± 5.96
Bemcentinib	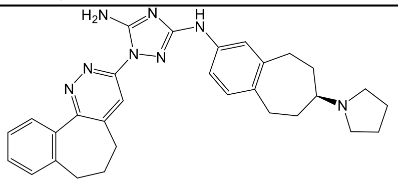	Repurposed	Catalytic Palm Thumb	18.47 ± 3.41
Theaflavin 3-gallate	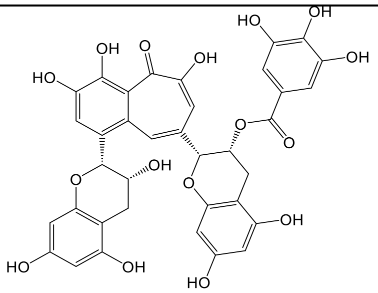	Natural	Catalytic	19.8 ± 3.49

Beta-Boswellic acid	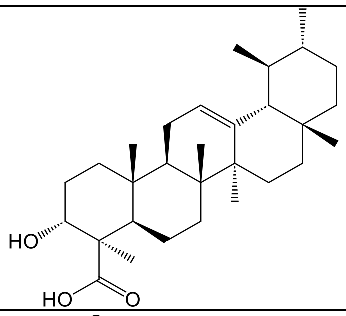	Natural	Palm Thumb	21.09 ± 4.45
BMS-986142	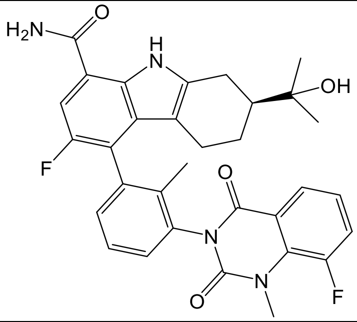	Repurposed	Catalytic Palm Thumb	22.82 ± 3.63
Lenrispodun	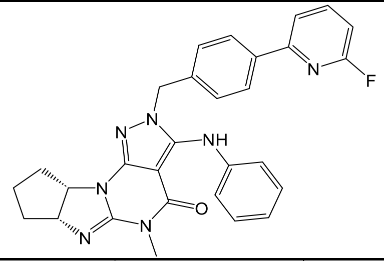	Repurposed	Thumb	24.20 ± 8.68
Pulchinenoside A	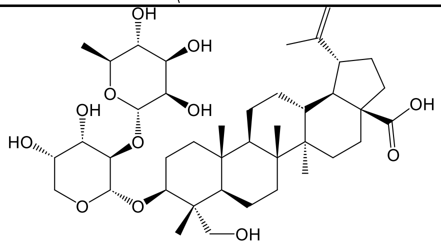	Natural	Catalytic	24.69 ± 0.42
Ursonic acid	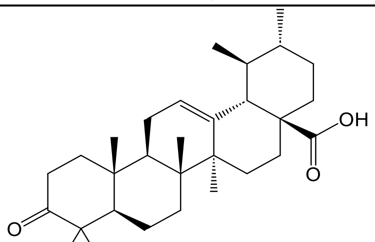	Natural	Palm Thumb	26.54 ± 12.61
Avatrombopag	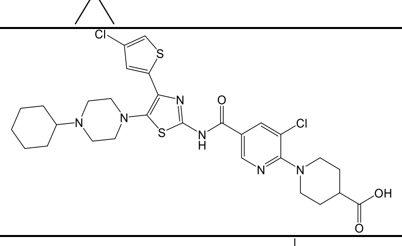	Repurposed	Catalytic Palm	27.16 ± 7.35
(1S,9R)-11-[2-(2,5,9-trimethyl-7-oxo-3-phenylfuro[3,2-g]chromen-6-yl)acetyl]-7,11-diazatricyclo[7.3.1.02,7]trideca-2,4-dien-6-one	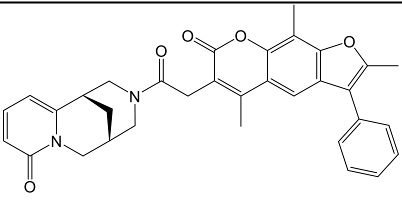	Natural	Palm Thumb	28.27 ± 6.24

3-methyl-8-[(2-methylphenyl)iminomethyl]-5-propan-2-yl-2-[1,6,7-trihydroxy-3-methyl-8-[(2-methylphenyl)iminomethyl]-5-propan-2-ylnaphthalen-2-yl]naphthalene-1,6,7-triol	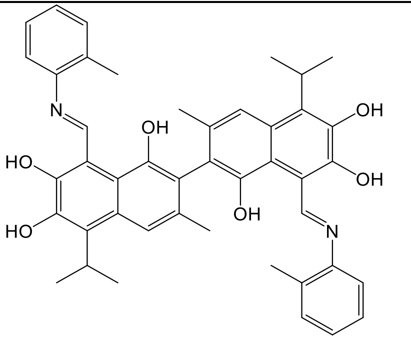	Natural	Catalytic Palm Thumb	28.44 ± 7.42
Enoxolone	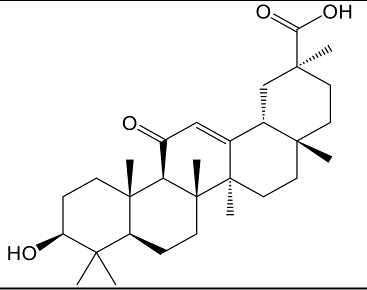	Repurposed	Catalytic Palm Thumb	35.5 ± 4.61
Kuwanon G	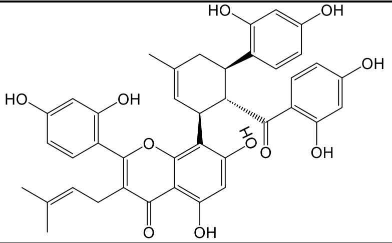	Natural	Palm	41.0 ± 6.18
Paritaprevir	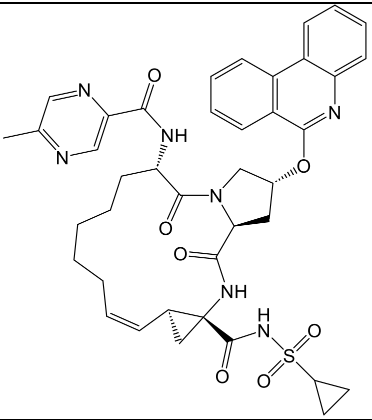	Repurposed	Thumb	42.23 ± 9.97
(7S,9E,11S,12S,13S,14S,15S,16R,17S,18S,19E,21Z)-2,13,15,17-tetrahydroxy-11-methoxy-3,7,12,14,16,18,22-heptamethyl-26-pyrrolidin-1-yl-8,30-dioxa-24-azatetracyclo[23.3.1.14,7.05,28]triaconta-1(28),2,4,9,19,21,25-heptaene-	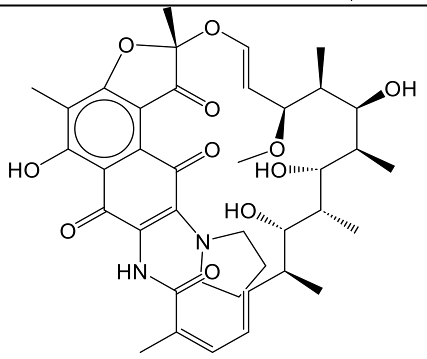	Natural	Catalytic Palm	46.11 ± 15.61

6,23,27,29-tetrone				
Sotorasib	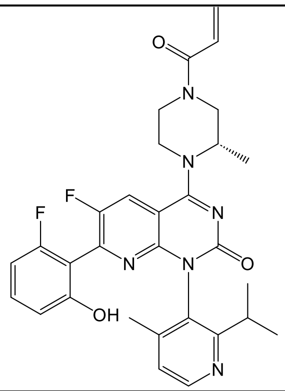	Repurposed	Catalytic<br>Palm<br>Thumb	47.0 ± 10.6
Cucurbitacin E	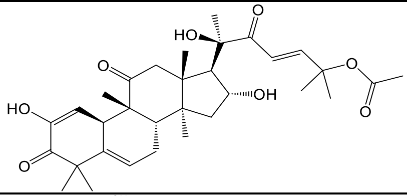	Natural	Palm	47.24 ± 20.49
Pseudoginsenoside Rt1	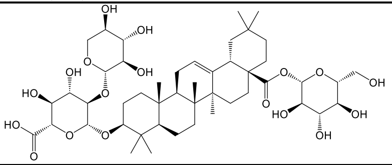	Natural	Palm	49.25 ± 5.86
(2S,4aS,6aR,6aS,6bR,8aR,10S,12aS,14bR)-10-hydroxy-2,4a,6a,6b,9,9,12a,14b-octamethyl-13-oxo-N-propyl-1,3,4,5,6,6a,7,8,8a,10,11,12-dodecahydropicene-2-carboxamide	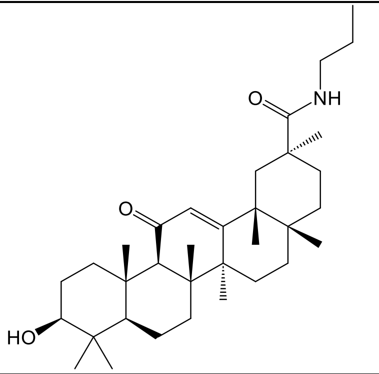	Natural	Palm<br>Thumb	50.20 ± 15.13
Tomatidine	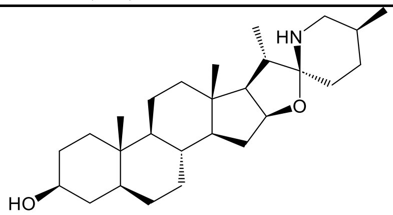	Natural	Palm	51.36 ± 5.58
Saikosaponin B2	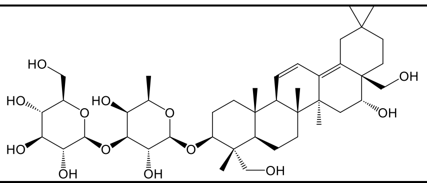	Natural	Thumb	54.55 ± 13.62
Carbenoxolone	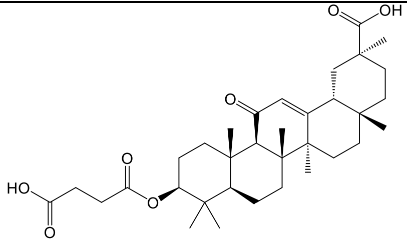	Repurposed	Catalytic<br>Palm	55.8 ± 8.66

Vidupiprant	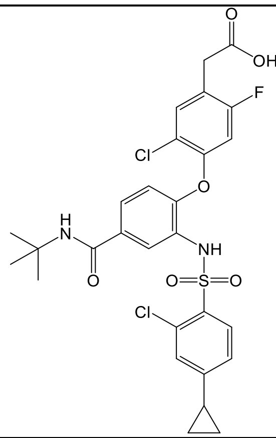	Repurposed	Catalytic	63.51 ± 24.28
Kuwanon H	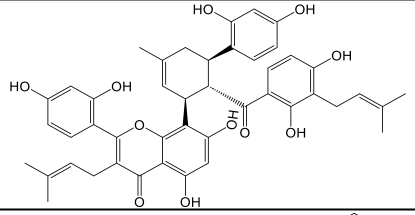	Natural	Palm	66.18 ± 1.81
2-(2-oxo-4-phenylchromen-7-yl)oxy-N-(4-phenylmethoxyphenyl)acetamide	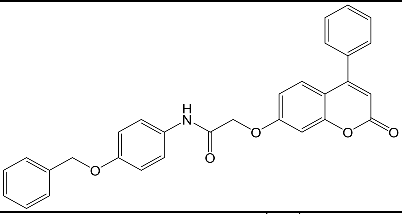	Natural	Palm	80.54 ± 6.24
Quillaic acid	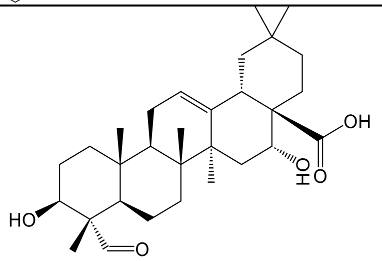	Natural	Palm	83.89 ± 9.31
3-[[(E)-2-[(4-benzamidobenzoyl)amino]-3-phenylprop-2-enoyl]amino]benzoic acid	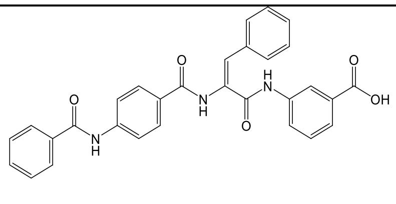	Natural	Thumb	85.70 ± 0.62
SSR240612	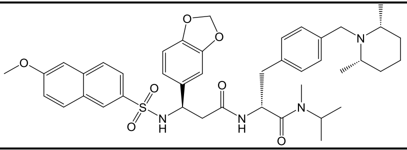	Repurposed	Catalytic	85.85 ± 4.35
Echinocystic acid	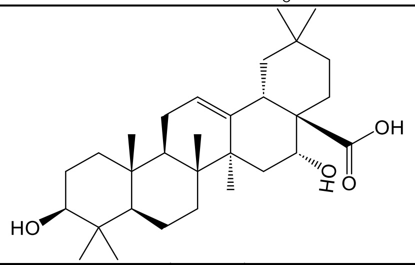	Natural	Palm	88.54 ± 16.21
N-[(E)-3-[[3-[[(E)-2-benzamido-3-(furan-2-yl)prop-2-enoyl]amino]-	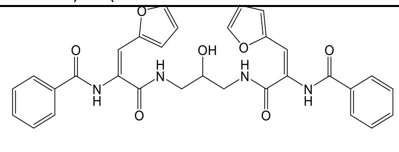	Natural	Palm	90.13 ± 13.96

2-hydroxypropyl]amino]-1-(furan-2-yl)-3-oxoprop-1-en-2-yl]benzamide				
HMR1031	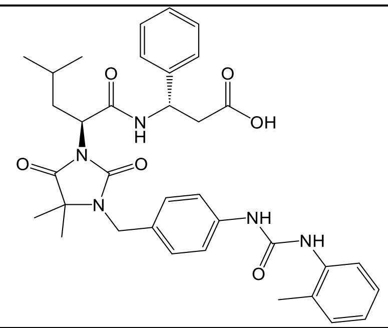	Repurposed	Catalytic Palm Thumb	91.15 ± 4.88
Alpha-Boswellic acid	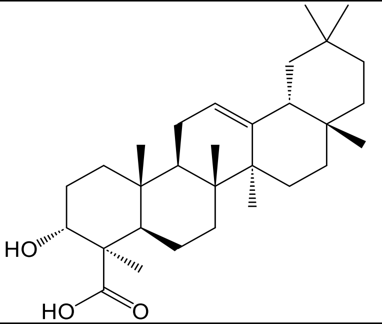	Natural	Catalytic Palm Thumb	93.54 ± 9.14
Rifaximin	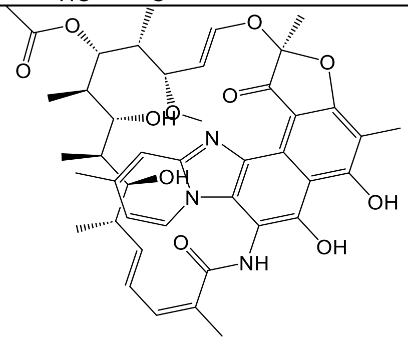	Repurposed	Catalytic	>100
Cucurbitacin D	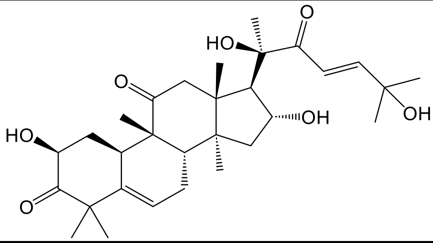	Natural	Palm	>100
Ginsenoside F1	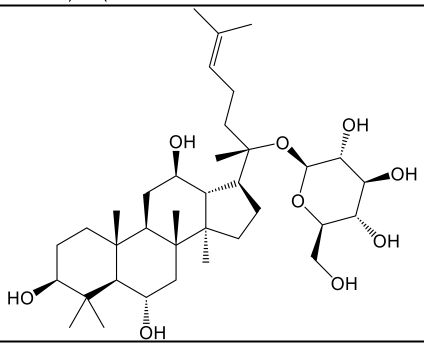	Natural	Palm	>100

20(R)-Ginsenoside Rh2	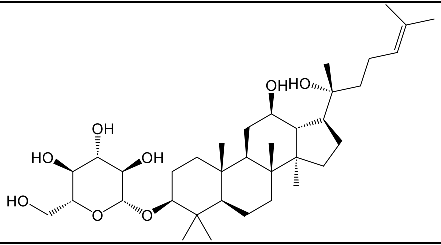	Natural	Palm	>100
Temoporfin	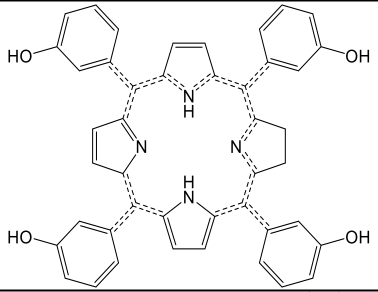	Repurposed	Catalytic	>100
Ecliptasaponin A	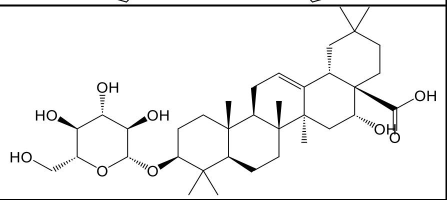	Natural	Palm	>100
Fenretinide	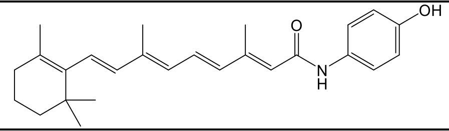	Repurposed	Thumb	>100
Lupeol	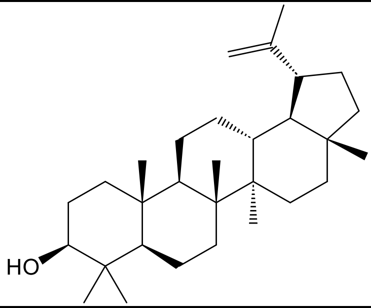	Natural	Thumb<br>Palm	>100
Smilagenin	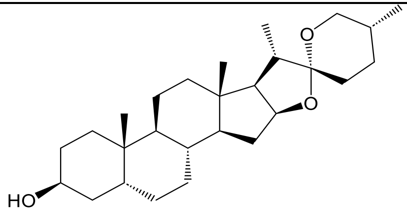	Repurposed	Thumb	>100
Lupenone	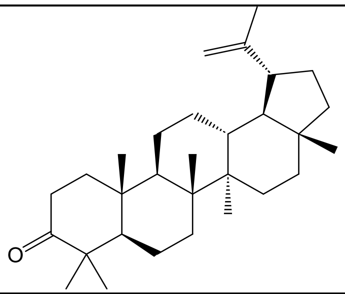	Natural	Palm	>100
Alpha-Spinasterol glucoside	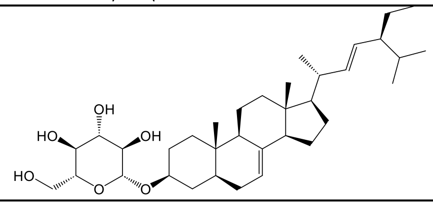	Natural	Palm	>100

Hellebrin	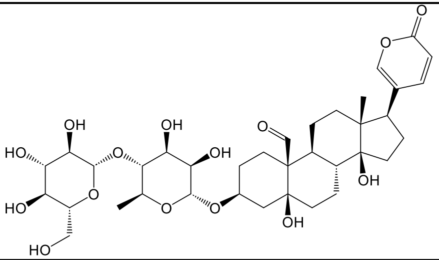	Natural	Palm	>100
N-[3-chloro-4-[(3-fluorophenyl)methoxy]phenyl]-6-[(*E*)-*N*-[[(3*R*)-morpholin-3-yl]methoxy]-*C*-prop-1-ynylcarbonimidoyl]quinazolin-4-amine	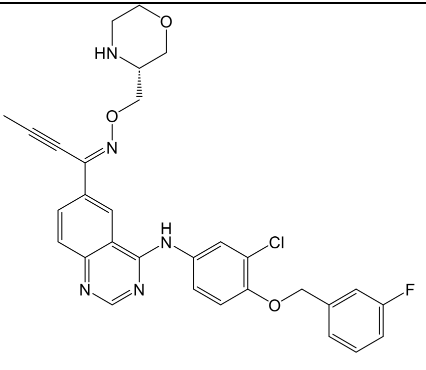	Repurposed	Palm	>100
Ursolic acid	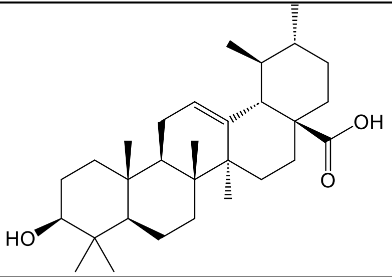	Repurposed	Catalytic Palm Thumb	>100
(1*S*,2*Z*,5*R*)-2-[1-[4-[4-[[(1*Z*)-1-[(1*S*,5*R*)-6,6-dimethyl-3-oxo-2-bicyclo[3.1.0]hexanylidene]ethyl]amino]phenoxy]anilino]ethylidene]-6,6-dimethylbicyclo[3.1.0]hexan-3-one	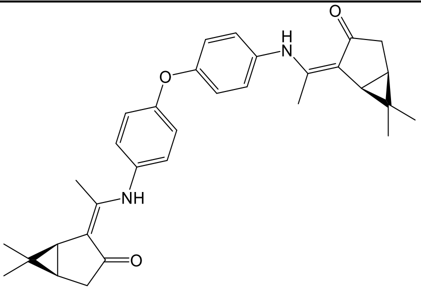	Natural	Palm	>100
Taladegib	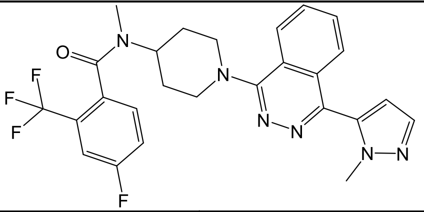	Repurposed	Catalytic Palm Thumb	>100
Eganelisib	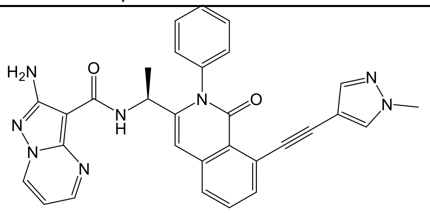	Repurposed	Catalytic Palm	>100

Cycloastragenol	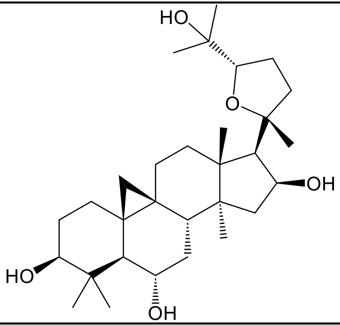	Natural	Palm<br>Thumb	>100
Ginsenoside Rg5	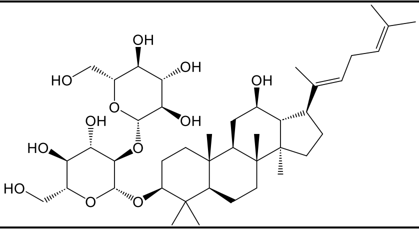	Natural	Thumb	>100
Picfeltarracemin Ia	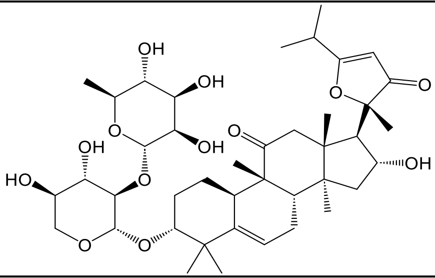	Natural	Catalytic<br>Palm	>100
13-{3-[4-(dibenzo[b,f][1,4]oxazepin-11-yl)piperazin-1-yl]-3-oxopropyl}-8,13-dihydroindolo[2',3':3,4]pyrido[2,1-b]quinazolin-5(7H)-one	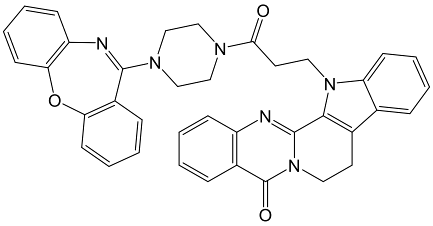	Natural	Palm	>100
Lixivaptan	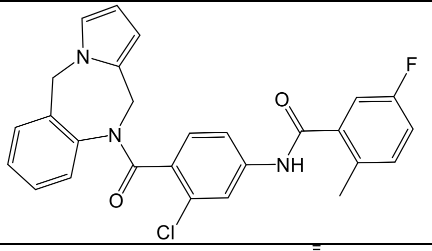	Repurposed	Thumb	>100
Tormentic acid	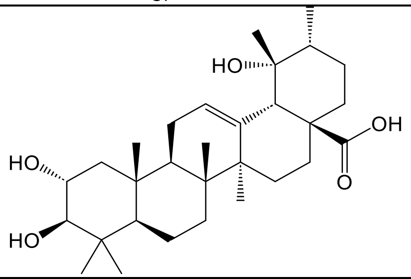	Natural	Catalytic<br>Palm<br>Thumb	>100

Adavivint	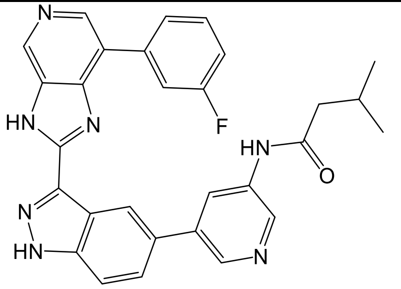	Repurposed	Palm<br>Thumb	>100
Omilancor	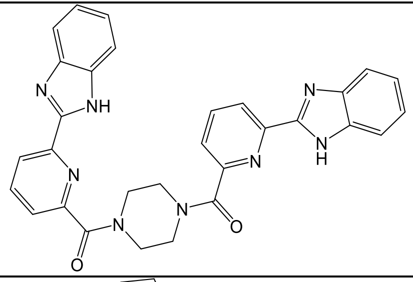	Repurposed	Catalytic<br>Palm	>100
Bromocryptine	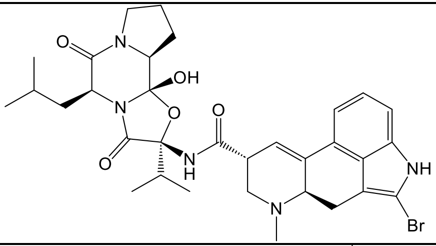	Repurposed	Palm<br>Thumb	>100
Vorapaxar	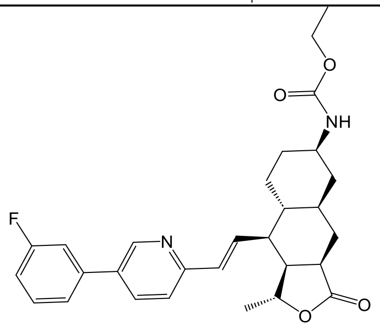	Repurposed	Catalytic	>100
Pachymic acid	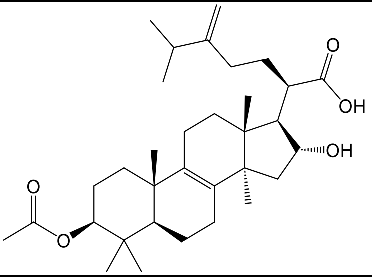	Natural	Palm	>100
Linsitinib	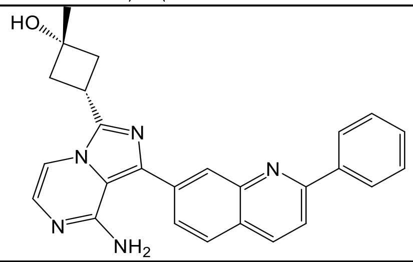	Repurposed	Palm	>100

Corosolic acid	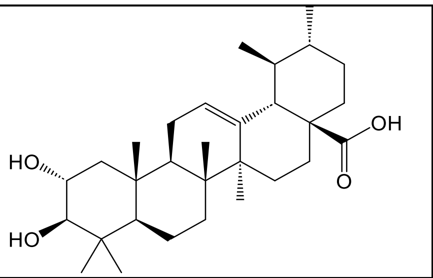	Repurposed	Catalytic Palm Thumb	>100
Prosapogenin A	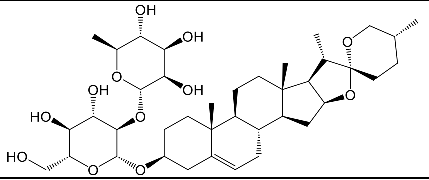	Natural	Palm	>100
3-Dehydrotrametenolic acid	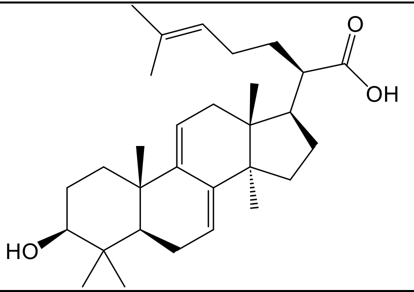	Natural	Palm	>100
Lifitegrast	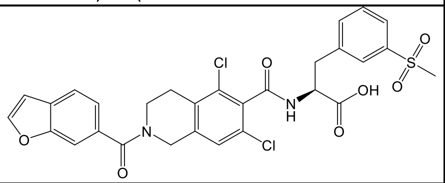	Repurposed	Palm	>100
(20R)-Protopanaxadiol	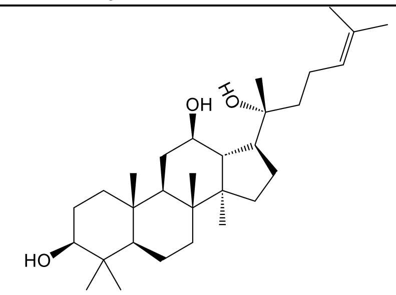	Natural	Palm	>100
Asiatic Acid	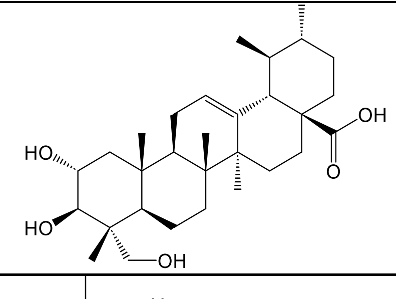	Natural	Palm	>100
(1'S,3R,3'S,7a'S)-5",7"-dimethyl-1'-{[3-(prop-2-en-1-yloxy)phenyl]carbonyl}-5',6',7',7a'-tetrahydro-1'H-dispiro[indole-3,2'-pyrrolizine-3',3"-indole]-2,2"(1H,1"H)-dione	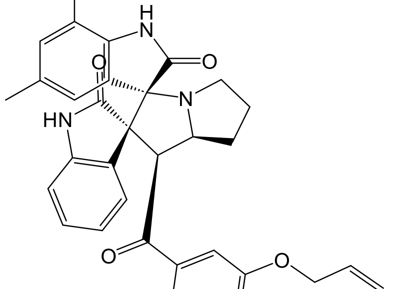	Natural	Thumb	>100

Senegenin	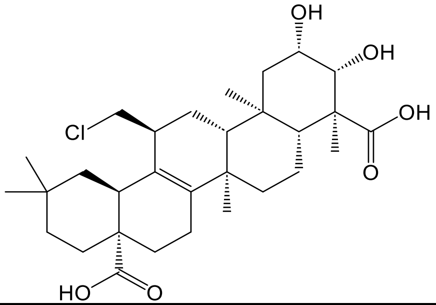	Natural	Catalytic	>100
(2S,3R,4R,5R,6S)-2-[(2R,3R,4S,5S,6R)-4,5-dihydroxy-6-(hydroxymethyl)-2-[(1R,2S,4S,5'R,6R,7S,8S,9S,12S,13R,16S)-8-hydroxy-5',7,9,13-tetramethylspiro[5-oxapentacyclo[10.8.0.02,9.04,8.013,18]icos-18-ene-6,2'-oxane]-16-yl]oxyoxan-3-yl]oxy-6-methyloxane-3,4,5-triol	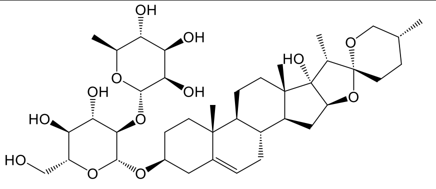	Natural	Palm	>100
Merestinib	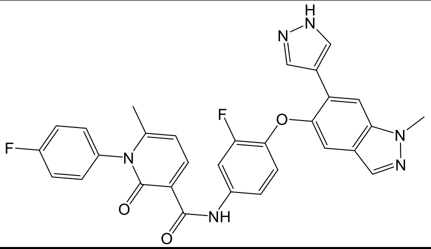	Repurposed	Palm	>100
Madecassic Acid	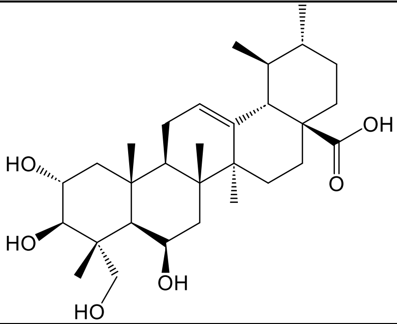	Natural	Palm	>100
Danicopan	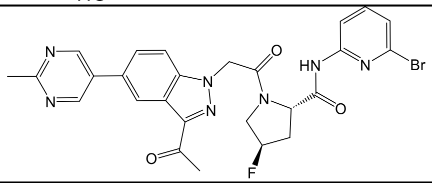	Repurposed	Catalytic	>100

6,10,11-Hexol	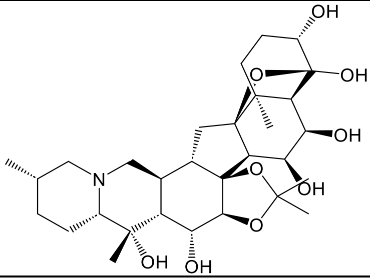	Natural	Palm	>100
Sograzepide	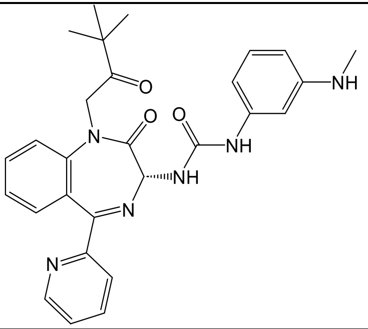	Repurposed	Catalytic	>100
Alisol C monoacetate	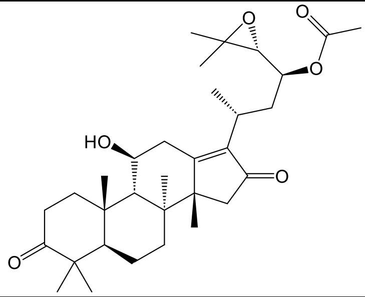	Natural	Palm	>100
Tradipitant	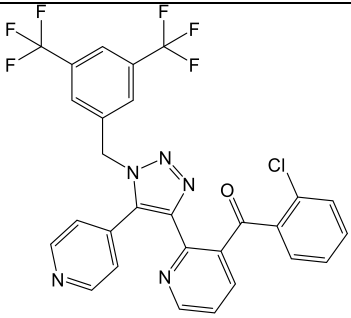	Repurposed	Palm Thumb	>100
Umbralisib	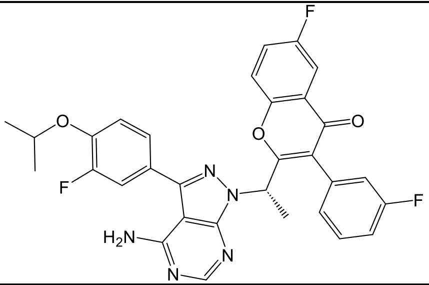	Repurposed	Catalytic Palm Thumb	>100
Betulinaldehyde	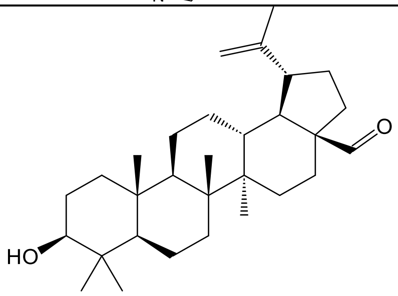	Natural	Palm	>100

Pseudoginsenoside Rt5	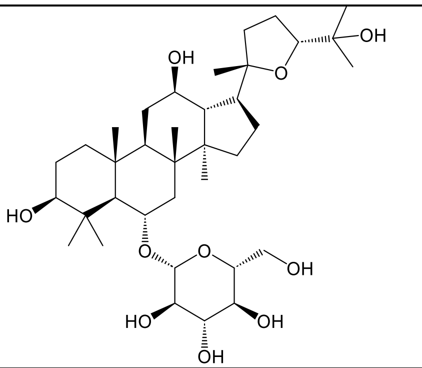	Natural	Palm Thumb	>100
Mogroside IIe	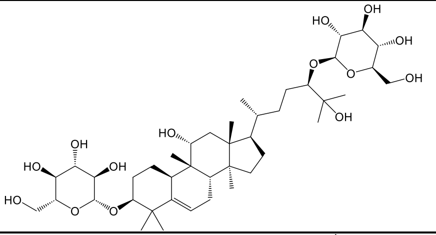	Natural	Catalytic	>100
(20R)-Ginsenoside Rh1	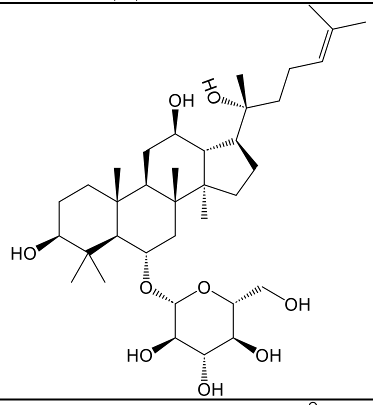	Natural	Palm	>100
3,29-Dibenzoyl Rarounitriol	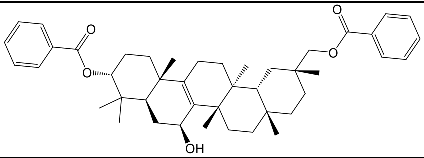	Natural	Catalytic	>100
Cyasterone	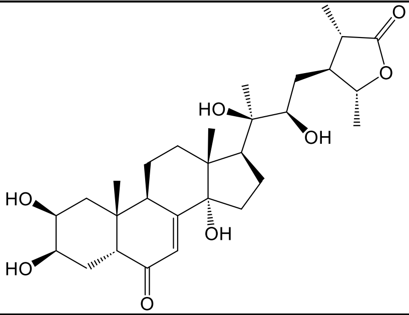	Natural	Palm	>100
Saikosaponin D	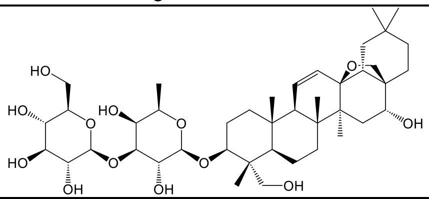	Natural	Catalytic	>100

Imperialine 3-beta-D-glucoside	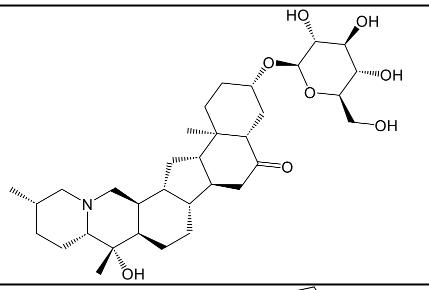	Natural	Palm	>100
Avacopan	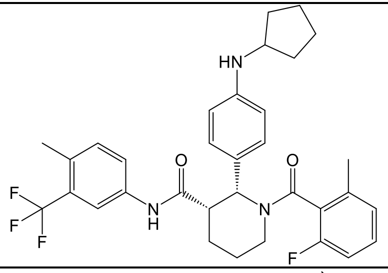	Repurposed	Catalytic Thumb	>100
(20S)-Protopanaxadiol	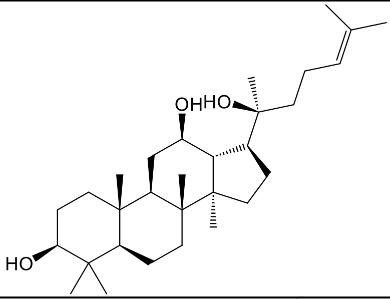	Natural	Palm	>100
Ecdysterone 2,3:20,22-diacetonide	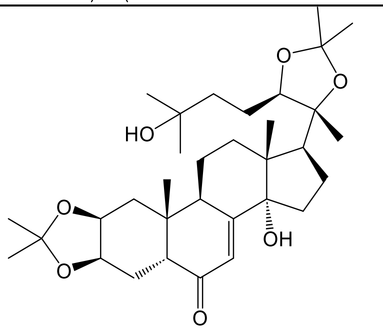	Natural	Palm Thumb	>100
Dutasteride	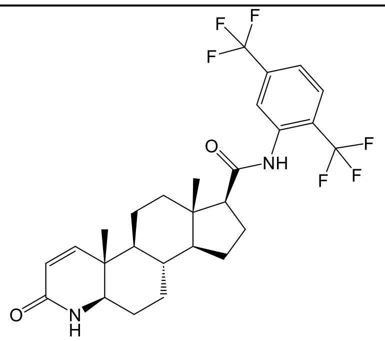	Repurposed	Thumb	>100
20-Hydroxyecdysone 20,22-acetonide	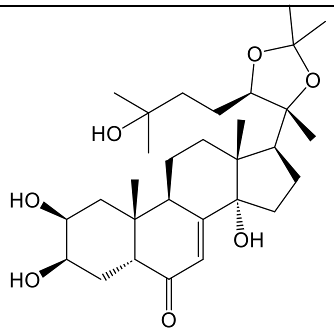	Natural	Palm	>100

(2R)-[(N-{[1-(tert-butoxycarbonyl)-4-phenylpiperidin-4-yl]carbonyl}-D-valyl)amino](phenyl)ethanoic acid	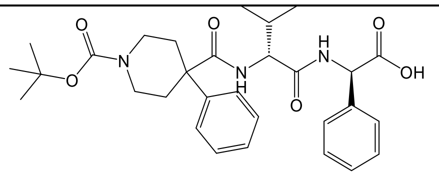	Natural	Palm	>100
Gsk-2838232	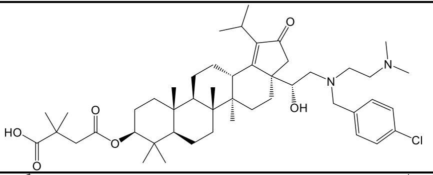	Repurposed	Thumb	>100
Fenebrutinib	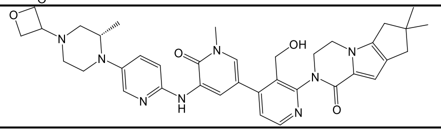	Repurposed	Palm	>100
Rotundic acid	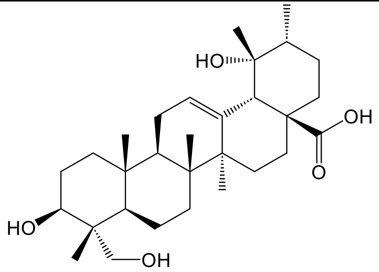	Natural	Palm	>100
Vodobatinib	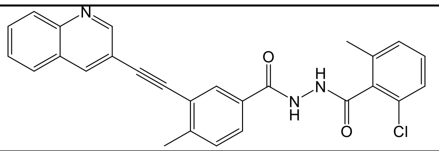	Repurposed	Catalytic Palm	>100
Monensin	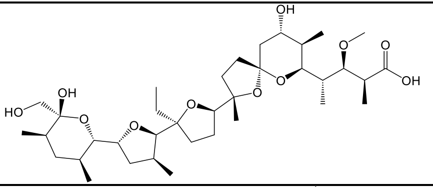	Natural	Palm	>100
Lupeol Acetate	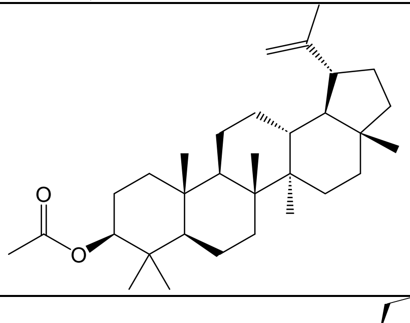	Natural	Palm	>100
Daucosterol	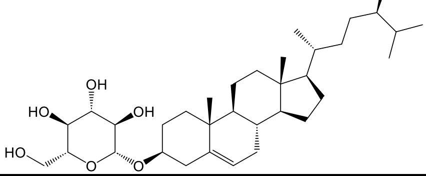	Repurposed	Catalytic Thumb	>100

Pomolic acid	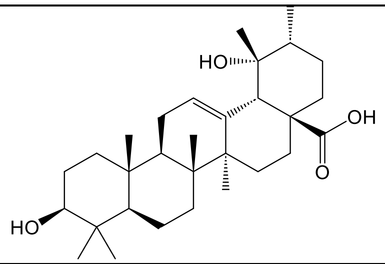	Natural	Palm<br>Thumb	>100
Disogluside	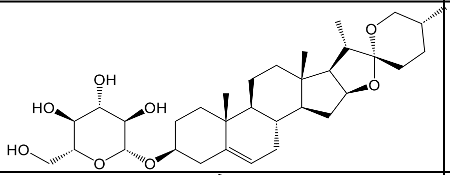	Natural	Palm	>100
Doramapimod	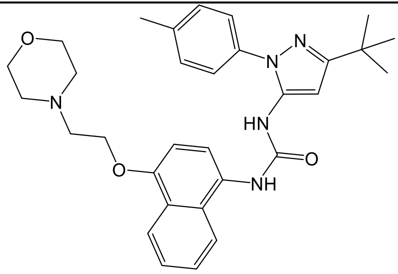	Repurposed	Palm	>100
(2S,4aS,6aR,6aS,6bR,8aR,10S,12aS,14bR)-10-hydroxy-2,4a,6a,6b,9,9,12a,14b-octamethyl-N-(4-methylphenyl)-13-oxo-1,3,4,5,6,6a,7,8,8a,10,11,12-dodecahydropicene-2-carboxamide	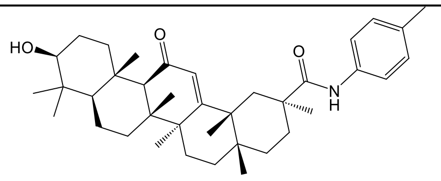	Natural	Catalytic<br>Palm<br>Thumb	>100
Tucatinib	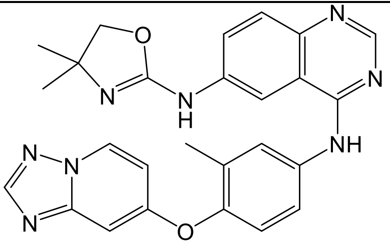	Repurposed	Catalytic	>100
Wilforlide A	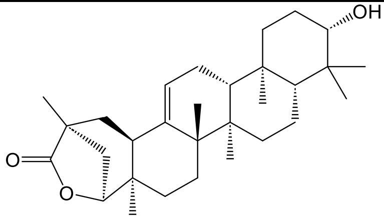	Natural	Catalytic<br>Palm	>100
Peimisine	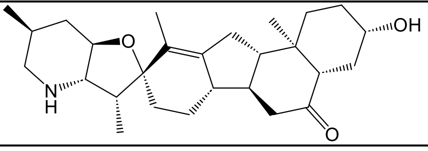	Natural	Palm	>100

Maslinic Acid	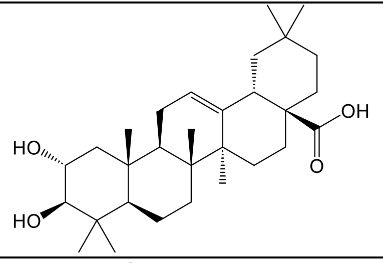	Natural	Palm Thumb	>100
Hypericin	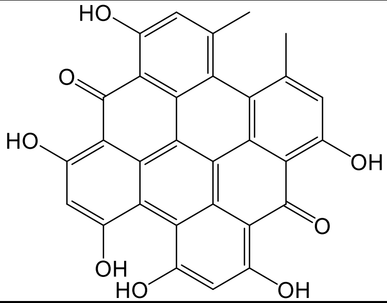	Repurposed	Catalytic	Fluorescence interference
Succinobucol	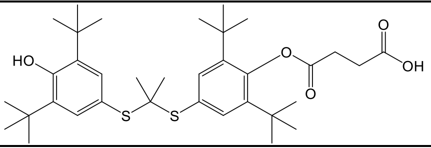	Repurposed	Catalytic	Fluorescence interference
Simeprevir	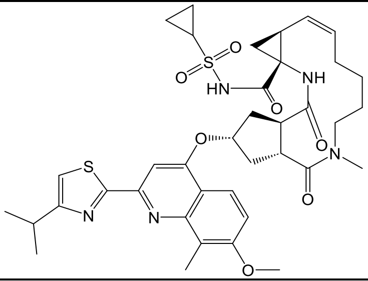	Positive control (Lo et al., 2021)	-	9.37 ± 3.31

[a]Compound concentration required to inhibit by 50% the SARS-CoV-2 RTC enzymatic activity. Data represent the mean and SD of at least 3 independent experiments

Figure S1: Predicted interaction of AKBA with SARS-CoV-2 nsp12

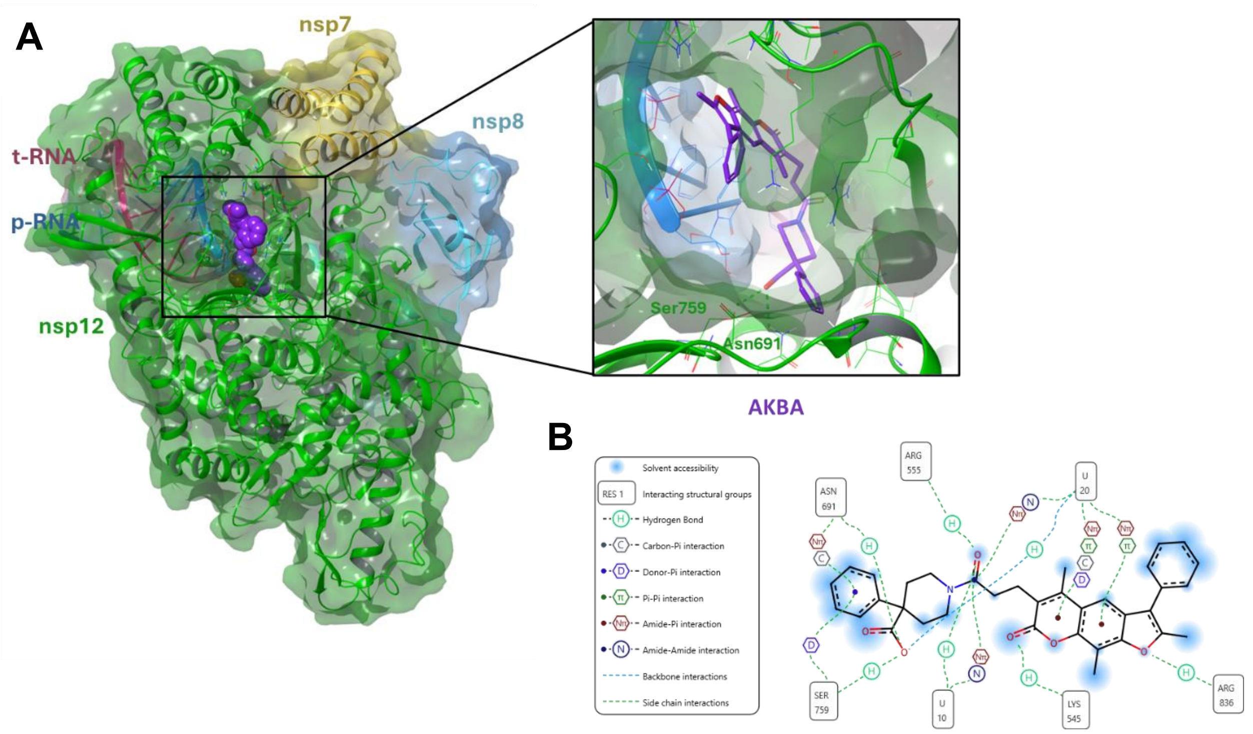

Fig. S1. *(A) Binding pose of AKBA in the catalytic site of SARS-CoV-2 nsp12 in presence of RNA. (B) Schematic representation of the predicted interactions of AKBA with the amino acids of the catalytic site. AKBA binds in the catalytic active site of nsp12 making polar interactions into a hydrophilic pocket, in particular with the residues Asn691 and Ser759, as also showed in the binding mode of the known inhibitor remdesivir-DP (Fig. 4).*

Figure S2: Predicted interaction of Cmp_4 with SARS-CoV-2 nsp12

A

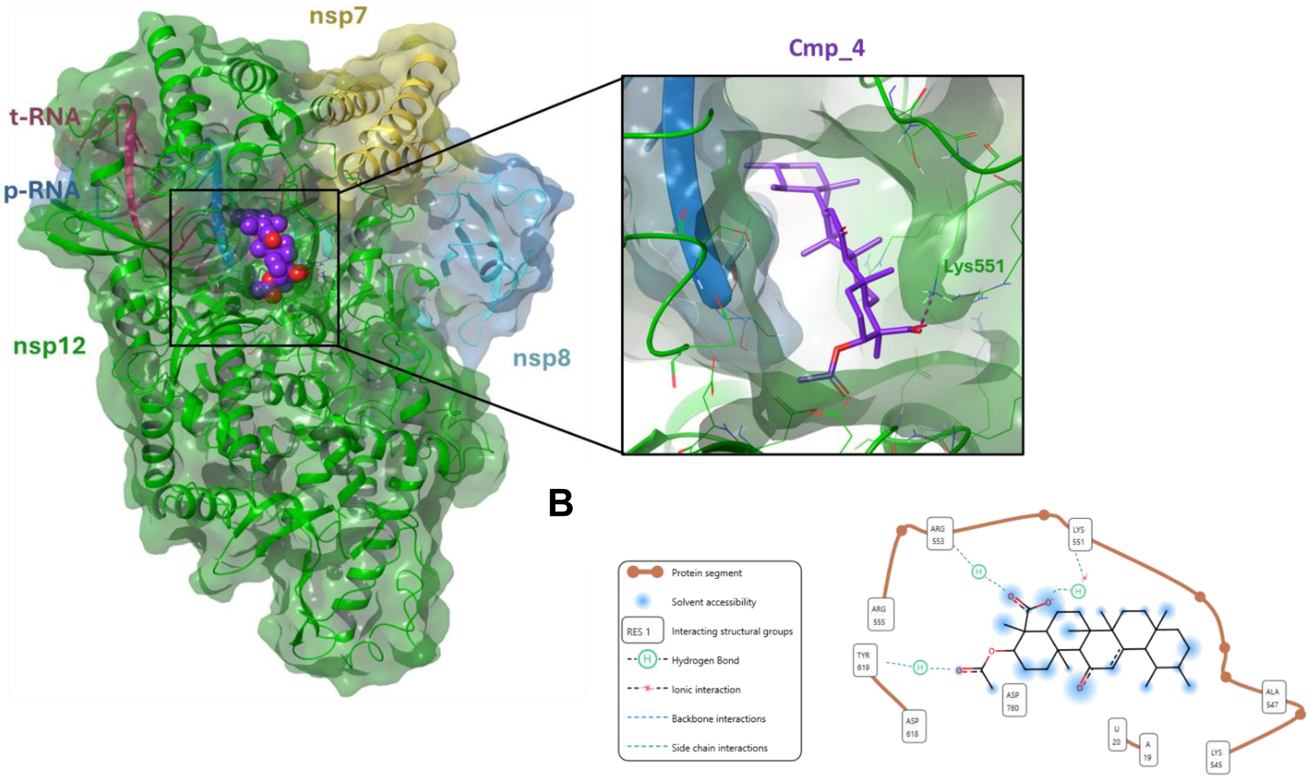

Fig. S2. *(A) Binding pose of Cmp_4 in the catalytic site of SARS-CoV-2 nsp12 in presence of RNA. (B) Schematic representation of the predicted interactions of Cmp_4 with the amino acids of the catalytic site. Cmp_4 binds in the catalytic active site of nsp12 making in particular interactions with the key residue Lys551 lining the NTP entry channel.*

Figure S3: Binding check of SARS-CoV-2 RTC and venetoclax

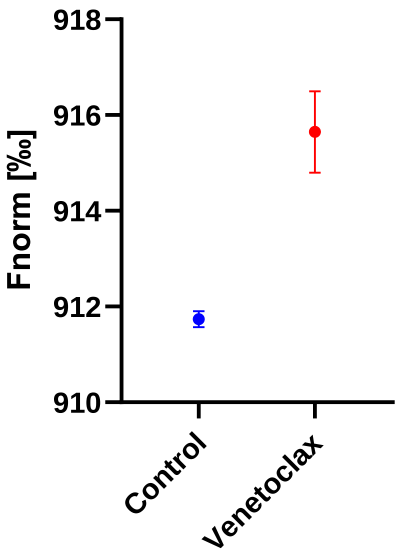

Fig. S3. *Binding check of venetoclax (500 µM) and SARS-CoV-2 minimal RTC, as determined by Microscale Thermophoresis. Fnorm[‰] expresses the ratio between the fluorescence values before and after laser activation. Obtained signal-to-noise ratio was 7.7, and the response amplitude was 3.9.*

Figure S4: BOILED-Egg plot of top-ranked compounds

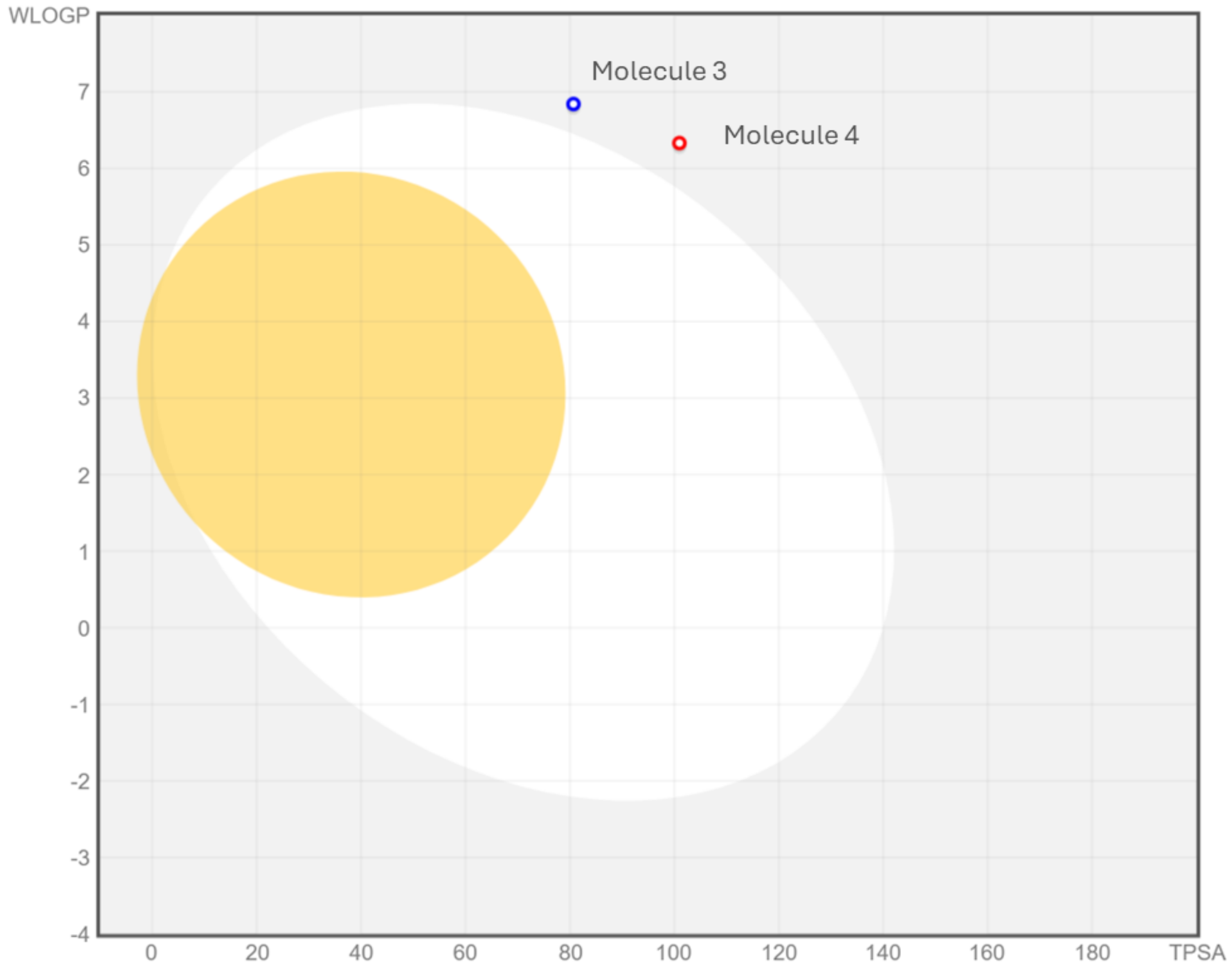

Fig. S4. *BOILED-Egg plot of molecule 3 (AKBA) and molecule 4 (Cmp_4), based on SwissADME predictions. Rose bengal and venetoclax were excluded from the plot due to exceeding the model's structural limits (MW > 800 Da and/or TPSA > 140 Å²).*
